# Exploring Short-Wavelength Phase-Matching Nonlinear Optical Crystals by Employing KBe_2_BO_3_F_2_ as the Template

**DOI:** 10.1021/acscentsci.2c00832

**Published:** 2022-11-11

**Authors:** Zijian Li, Wenqi Jin, Fangfang Zhang, Zhihua Yang, Shilie Pan

**Affiliations:** Research Center for Crystal Materials, CAS Key Laboratory of Functional Materials and Devices for Special Environments, Xinjiang Technical Institute of Physics and Chemistry of CAS, 40-1 South Beijing Road, Urumqi 830011, China; Center of Materials Science and Optoelectronics Engineering, University of Chinese Academy of Sciences, Beijing 100049, China

## Abstract

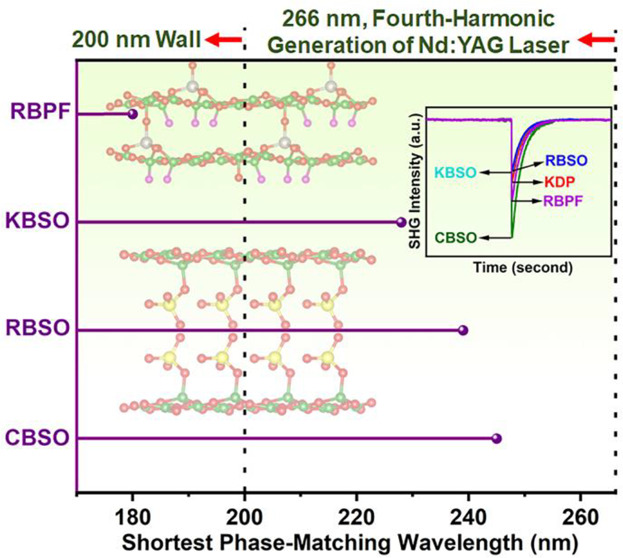

Exploration of nonlinear optical (NLO) crystals that are competent in generating short-wavelength ultraviolet (UV, λ ≤ 266 nm, and even deep-UV, λ ≤ 200 nm) coherent light output by direct second harmonic generation (SHG) remains a formidable challenge. Herein, four UV/deep-UV NLO crystals, M_2_B_4_SO_10_ (M = K, Rb, and Cs) and Rb_3_B_11_PO_19_F_3_, were successfully synthesized by evolving the KBe_2_BO_3_F_2_ (KBBF) structure into mixed-anionic borosulfate and fluoroborophosphate systems. They display functional [B_4_SO_10_]_∞_ or [B_11_PO_19_F_3_]_∞_ KBBF-type layers that are composed of [BO_3_], [BO_4_], and [SO_4_] groups or [BO_3_], [BO_4_], [BO_3_F], and [PO_4_] groups, respectively. Experimental characterization and numerical computation results indicate that these crystals possess exceptional NLO performance, including large SHG responses (0.9–1.7 × KDP at 1064 nm and 0.1–0.3 × β-BBO at 532 nm) and adequate birefringence to fulfill the SHG phase-matching (PM) condition at 266 nm. In particular, the shortest type-I PM wavelength (λ_PM_) of Rb_3_B_11_PO_19_F_3_ reaches 180 nm, which implies that Rb_3_B_11_PO_19_F_3_ can become a prospective deep-UV NLO crystal. In addition, single crystals of K_2_B_4_SO_10_, Rb_2_B_4_SO_10_, and Cs_2_B_4_SO_10_ are easily obtained by the high-temperature solution approach. This work will facilitate the discovery of short-wavelength PM NLO crystals by using the KBBF structure as the template.

## Abstract 2

A series of excellent short-wavelength ultraviolet NLO crystals were derived from KBBF through replacement of [Be_2_BO_3_F_2_]_∞_ functional layers in KBBF with amazing [B_4_SO_10_]_∞_ and [B_11_PO_19_F_3_]_∞_ layers.

## Introduction

The frequency doubling technique of the solid-state lasers through nonlinear optical (NLO) crystals, for instance, fourth-harmonic generation (FHG) of an Nd:YAG laser to produce ultraviolet (UV, λ ≤ 266 nm) or even deep-UV (λ ≤ 200 nm) laser coherent light sources, has pivotal applications in modern scientific equipment,^1−7^ such as ultrahigh energy resolution photoelectron spectroscopy and photoelectron emission microscopy as well as fields of semiconductor lithography, micro- and nano-fine-processing, etc.^8−13^ To date, a limited number of practically applicable materials are developed in the corresponding UV/deep-UV NLO systems, including KH_2_PO_4_ (KDP),^14^ LiB_3_O_5_ (LBO),^15^ CsLiB_6_O_10_ (CLBO),^16^ β-BaB_2_O_4_ (β-BBO),^17^ KBe_2_BO_3_F_2_ (KBBF),^18^ etc. With the development of short-wavelength UV laser science, there remains a pressing need to find new NLO crystals capable of producing UV/deep-UV coherent light via direct frequency doubling.^19,20^

At present, in crystals applied to all-solid-state lasers, coherent light with wavelengths less than 200 nm can only be generated by KBBF crystals through direct second harmonic generation (SHG).^21^ Extensive experimental and theoretical analyses suggest that the extraordinary properties of KBBF stem from the distinctive two-dimensional (2D) [Be_2_BO_3_F_2_]_∞_ functional layer in which the microscopic NLO-active [BO_3_] triangles feature a parallel alignment.^22,23^ Nevertheless, the process of crystal growth of KBBF inevitably uses highly toxic beryllium oxide which damages the environment and human body, and the feeble ionic K–F bonds among neighboring [Be_2_BO_3_F_2_]_∞_ layers hamper the large-sized crystal growth of KBBF.^24−28^ To overcome these issues, the [BeO_3_F] tetrahedral units in KBBF were replaced by [AlO_3_F], [AlO_4_], [ZnO_4_], [ZnO_3_X] (X = F, Cl, and Br), or [BO_3_F] tetrahedra units leading to the finding of a mass of KBBF-analogy borate NLO crystals, such as K_2_Al_2_B_2_O_7_,^29^ β-Rb_2_Al_2_B_2_O_7_,^30^ CsAlB_3_O_6_F,^31^ K_3_M_3_Li_2_Al_4_B_6_O_20_F (M = Sr and Ba),^32,33^ Cs_3_Zn_6_B_9_O_21_,^34^ AZn_2_BO_3_X_2_ (A = NH_4_, K, Rb, and Cs; X = F, Cl, and Br),^35,36^ AB_4_O_6_F (A = NH_4_, Na, Rb, and Cs),^4,37−39^ and MB_5_O_7_F_3_ (M = Sr and Ca).^40,41^ These KBBF-analogy borate crystals not only display outstanding linear and nonlinear optical performance in the short-wavelength UV field but also effectively avoid toxicity and layered growth problems. It follows that employing the KBBF structure as a template is one of the most effective strategies to design UV/deep-UV crystals.

The mixed-anionic compounds are attractive hunting grounds for probing NLO crystals due to their interesting physiochemical properties which are usually associated with their structural varieties.^42−44^ Belonging to the mixed-anionic system, borosulfates and fluoroborophosphates are rich in crystal structures that are benefited from the interconnection of B–O groups with the [SO_4_] or [PO_4_] groups, respectively.^45−48^ However, the small optical anisotropy of the rigid tetrahedra leads to non-phase-matching (NPM) properties that limit their application as novel NLO crystals in the short-wavelength UV area.^49,50^ In the recent work from our group, an attempt was made to introduce planar π-conjugated [BO_3_] groups into these systems to optimize their birefringence, and two cases of boron-rich borosulfate and fluoroborophosphate UV/deep-UV NLO crystals were successfully obtained.^8,44^ Nevertheless, comprehensive research on the structure–properties relationships and a systematic exploration of these mixed-anionic borosulfates and fluoroborophosphates are still lacking. In this regard, by employing the KBBF structure as the template, we performed further research to explore promising unexampled UV/deep-UV NLO crystals in the borosulfate and fluoroborophosphate systems. Consequently, four novel borosulfate and fluoroborophosphate crystals, M_2_B_4_SO_10_ (M = K, Rb, and Cs) and Rb_3_B_11_PO_19_F_3_, were obtained successfully. They possess 2D [B_4_SO_10_]_∞_ and [B_11_PO_19_F_3_]_∞_ layers, respectively, both of which resemble the [Be_2_BO_3_F_2_]_∞_ layers in the structure KBBF. It is noteworthy that both M_2_B_4_SO_10_ and Rb_3_B_11_PO_19_F_3_ display strong SHG response (0.9–1.7 × KDP at 1064 nm and 0.1–0.3 × β-BBO at 532 nm), proper birefringence (*Δn* = 0.058–0.080 at 1064 nm), and short phase-matching wavelength (λ_PM_ < 266 nm). More particularly, the calculated shortest λ_PM_ of Rb_3_B_11_PO_19_F_3_ is about 180 nm and breaks the “200 nm wall”, which implies that Rb_3_B_11_PO_19_F_3_ is a prospective deep-UV NLO crystal. On top of that, comprehensive calculations were carried out that indicate that the excellent properties of this series of compounds come from the functional [B_4_SO_10_]_∞_ and [B_11_PO_19_F_3_]_∞_ KBBF-type layers.

## Results and Discussion

### Structures of M_2_B_4_SO_10_

**Figure 1:**
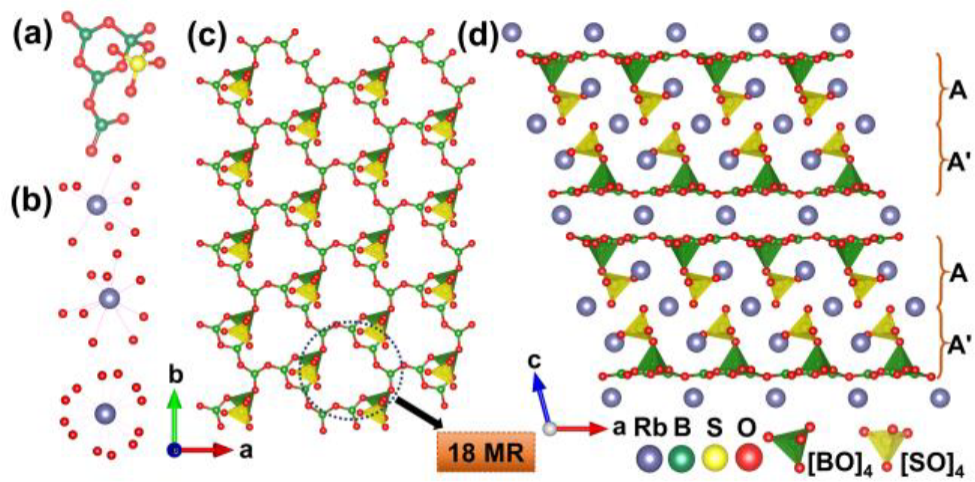
(a) [B_4_SO_12_] fundamental building blocks (FBBs) of M_2_B_4_SO_10_. (b) [RbO_8_], [RbO_9_], and [RbO_12_] polyhedra. (c) 2D [B_4_SO_10_]_∞_ layer constructed by FBBs. (d) Whole structure of Rb_2_B_4_SO_10_ viewing along the *b*-axis.

Since M_2_B_4_SO_10_ are isostructural and crystallize in a noncentrosymmetric monoclinic space group *C*2 (no. 5) (Table S1), Rb_2_B_4_SO_10_ is chosen as an example for crystal structure description. The asymmetric unit contains three crystallographically independent Rb atoms, four B atoms, one S atom, and ten O atoms (Table S2). Figure 1a shows that three [BO_3_] triangles, one [SO_4_] tetrahedron, and one [BO_4_] tetrahedron are linked to form the [B_4_SO_12_] fundamental building blocks (FBBs). The Rb^+^ cations cooperate with oxygen atoms to form [RbO_8_], [RbO_9_], and [RbO_12_] polyhedra (Figure 1b), separately. In the *ab*-plane, these FBBs are further bonded to form [B_4_SO_10_]_∞_ layers that contain large 18-membered rings (18-MRs) (Figure 1c). Notably, the corresponding bond lengths and angles of M_2_B_4_SO_10_ are similar to those of the reported borosulfates (Table S3–S5).^45,46^ Finally, along the *c*-axis, adjoining layers pile in reversely parallel with the −A′AA′A– order and are further connected through the Rb–O polyhedra through ionic bonds to establish the overall 3D framework (Figure 1d). Unsurprisingly, the unit cell axes and cell volumes become larger as the ionic radii of K, Rb, and Cs increase. The cell volume of the Cs-containing structure is significantly larger than those of the other structural analogues, but the increases along the *a*- and *b*-axes are nonobvious compared to the expansion along the *c*-axis; therefore, the structure accommodates larger alkali metals mainly by increasing the layer spacing along the *c*-axis (Table S1).

### Structure of Rb_3_B_11_PO_19_F_3_

**Figure 2:**
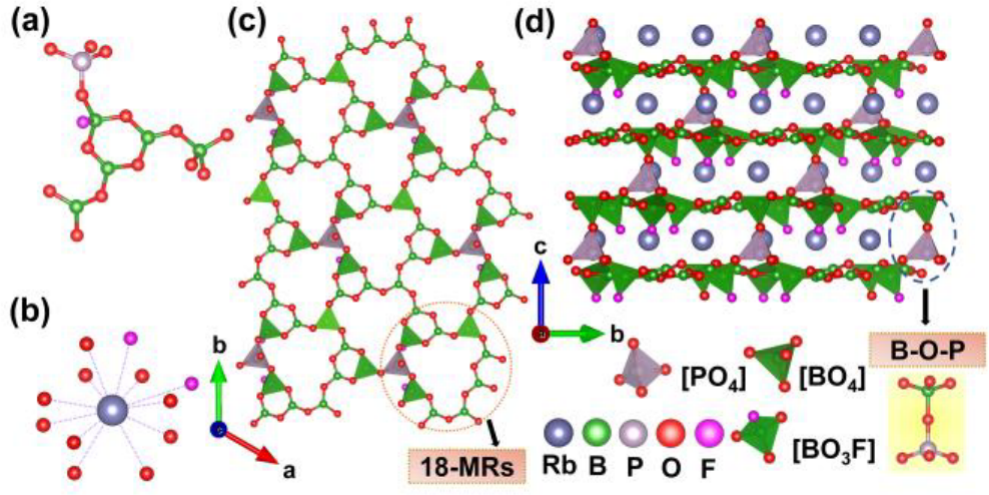
(a) [B_5_PO_14_F] FBBs of Rb_3_B_11_PO_19_F_3_. (b) [RbO_10_F_2_] polyhedron. (c) 2D [B_11_PO_19_F_3_]_∞_ layers with 18-MR channels. (d) Whole structure of Rb_3_B_11_PO_19_F_3_ viewing along the *a*-axis.

Rb_3_B_11_PO_19_F_3_ crystallizes in the acentric space group of *R*3 (no. 146) that vests in the trigonal crystal system (Table S1). In the structure, there exists one crystallographically independent Rb atom, one P atom, five B atoms, seven O atoms, and one F atom (Table S6). The B atoms coordinate with O or F atoms forming a [BO_3_] triangle, [BO_4_] tetrahedron, and [BO_3_F] tetrahedron, respectively. The [B_5_PO_14_F] FBBs of Rb_3_B_11_PO_19_F_3_ are composed of [BO_3_], [BO_4_], [BO_3_F], and [PO_4_] groups (Figure 2a). Meanwhile, the Rb^+^ cation connects with two F atoms and ten O atoms, forming a [RbO_10_F_2_] polyhedron (Figure 2b). The coplanar [BO_3_] triangles share O atoms with [BO_4_] and [BO_3_F] groups, which are ulteriorly linked by [PO_4_] tetrahedra to expand along the *ab*-plane to construct a 2D [B_11_PO_19_F_3_]_∞_ infinite layer (Figure 2c). It is important to note that the relevant bond lengths and angles are within a reasonable range (Table S7). Subsequently, these 2D [B_11_PO_19_F_3_]_∞_ layers heap along the *c*-axis (Figure 2d). Finally, these layers are attached by the B–O–P covalent bonding to establish a 3D framework; at the same time, the Rb^+^ cations are inset in the interlayers (Figure 2d).

### Structural Evolution

**Figure 3:**
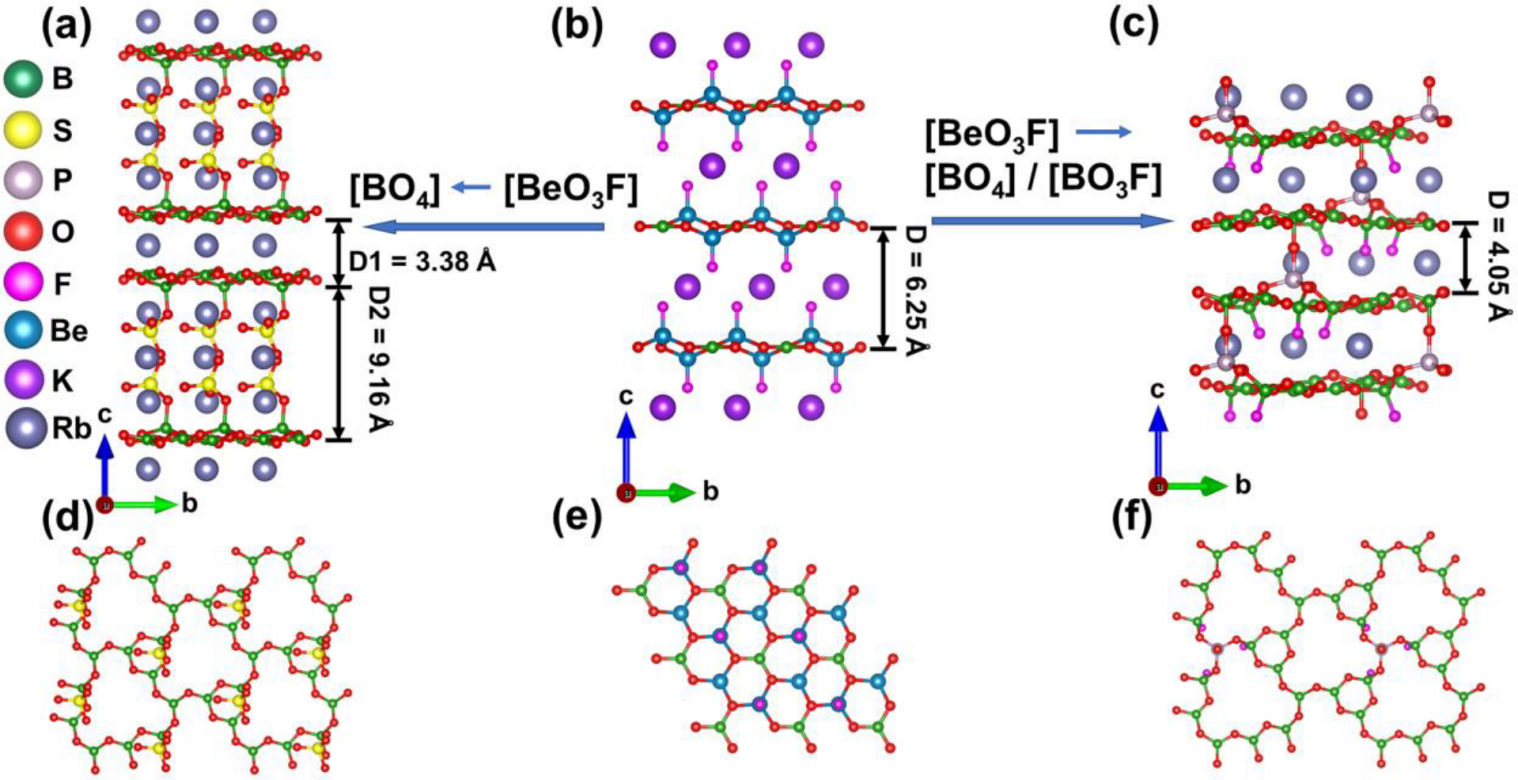
(a–c) Structural evolution from KBBF to M_2_B_4_SO_10_ and Rb_3_B_11_PO_19_F_3_. 2D [B_4_SO_10_]_∞_ (d), [Be_2_BO_3_F_2_]_∞_ (e), and [B_11_PO_19_F_3_]_∞_ (f) layers in M_2_B_4_SO_10_, KBBF, and Rb_3_B_11_PO_19_F_3_, respectively.

The structures of both M_2_B_4_SO_10_ and Rb_3_B_11_PO_19_F_3_ exhibit fascinating layers that evolve from KBBF (Figure 3). For these four compounds, the coplanar [BO_3_] groups are attached by [BO_4_] or [BO_3_F] groups. Therefore, the above connection mode can be seen as replacing [BeO_3_F] in the KBBF structure with [BO_4_] or [BO_3_F] groups. Meanwhile, the special linkages B–O–S or B–O–P of borosulfates or fluoroborophosphates are retained (Figure 3a–c), which leads to a series of KBBF-type crystal structures. It is critical to note that the use of highly toxic beryllium oxide is effectively avoided by the above substitution of tetrahedral groups. In particular, the interlayer bonding is enhanced, and the layer spacing of Rb_3_B_11_PO_19_F_3_ is reduced by the B–O–P linkage, which will be conducive to overcoming the layering growth tendency (Figure 3c). In M_2_B_4_SO_10_ and Rb_3_B_11_PO_19_F_3_, the [BO_3_] units are arranged in parallel which can be conducive to generating large SHG response and optical anisotropy (Figure 3d–f). Notably, the dangling bonds presented in the coplanar alignment of [BO_3_] groups are thereby effectively eliminated, which could increase the band gap of these crystals.

### Optical Property Studies

**Figure 4:**
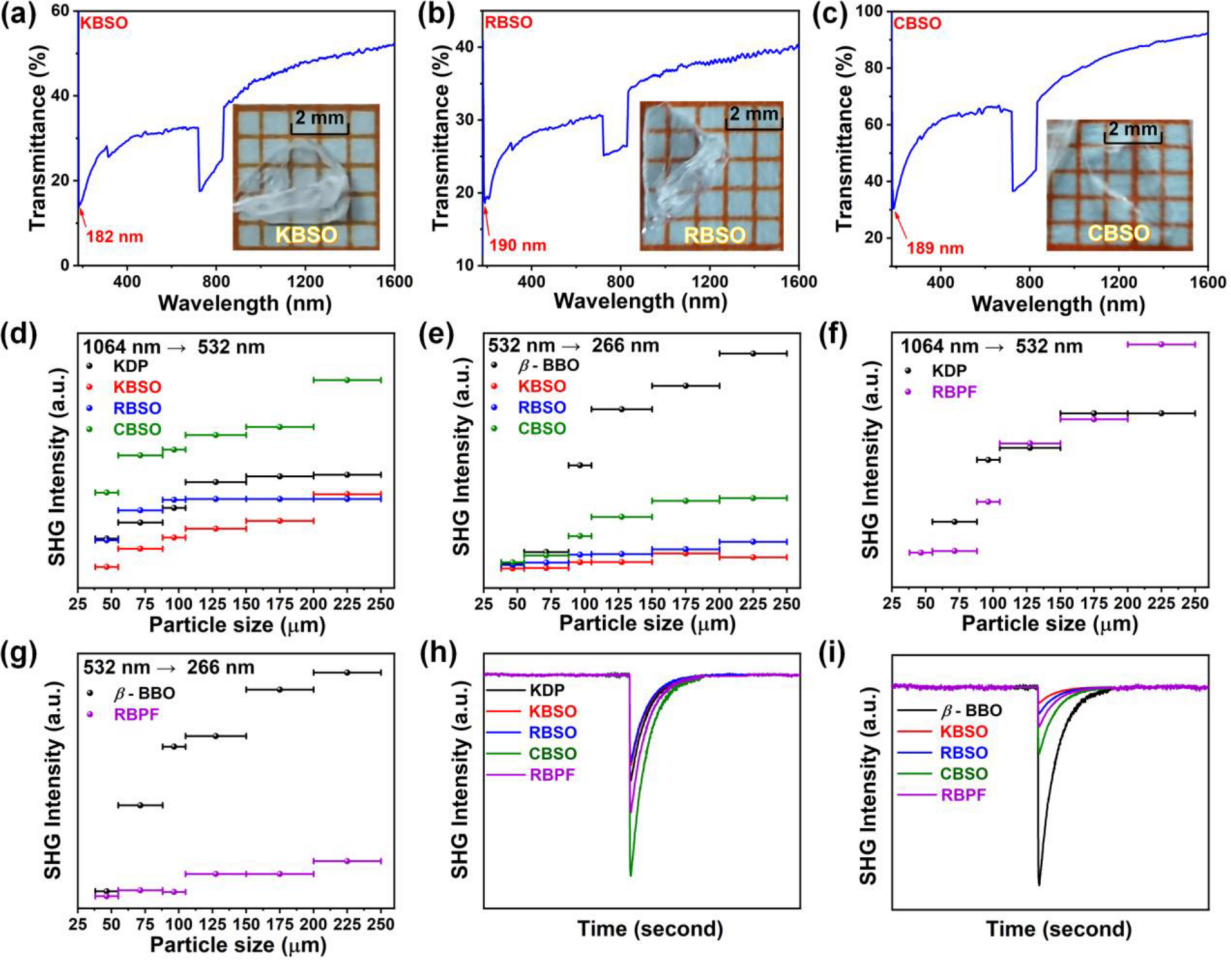
Transmittance spectra of K_2_B_4_SO_10_ (KBSO) (a), Rb_2_B_4_SO_10_ (RBSO) (b), and Cs_2_B_4_SO_10_ (CBSO) (c) in the region from 180 to 1600 nm. (d–g) Powder SHG measurements for K_2_B_4_SO_10_, Rb_2_B_4_SO_10_, Cs_2_B_4_SO_10_, and Rb_3_B_11_PO_19_F_3_ (RBPF) at 1064 and 532 nm with KDP and β-BBO working as the criteria, respectively. (h, i) Oscilloscope traces of the SHG signals for K_2_B_4_SO_10_, Rb_2_B_4_SO_10_, Cs_2_B_4_SO_10_, and Rb_3_B_11_PO_19_F_3_ powders (size range: 200–250 μm), respectively.

As presented in Figure S1, the UV–vis–NIR diffuse reflectance spectra of K_2_B_4_SO_10_, Rb_2_B_4_SO_10_, Cs_2_B_4_SO_10_, and Rb_3_B_11_PO_19_F_3_ were tested from 200 to 2600 nm, which display no significant absorption in the measured region. Meanwhile, single crystals of millimeter-sized K_2_B_4_SO_10_ and centimeter-sized Rb_2_B_4_SO_10_ and Cs_2_B_4_SO_10_ were obtained (Figure S2). To precisely obtain the UV absorption cutoff edge of these three crystals, transmittance spectroscopy tests were carried out using unpolished plates of the single crystals. As exhibited in Figure 4a–c, the transparent windows of all three crystals reach the deep-UV region, while at the same time, their cutoff edges can be observed to be 182, 189, and 190 nm, respectively. Their corresponding band gaps are 6.81, 6.53, and 6.56 eV, respectively. These values are not significantly different from that of (NH_4_)_2_B_4_SO_10_ (6.74 eV).^8^

### SHG Measurement

Since all four crystals reported in this paper are noncentric structures, the SHG effects of K_2_B_4_SO_10_, Rb_2_B_4_SO_10_, Cs_2_B_4_SO_10_, and Rb_3_B_11_PO_19_F_3_ were further investigated. When tested with 1064 and 532 nm laser sources, the SHG intensities of these four compounds increase continuously with increasing powder particle size, respectively. It is suggested that these four crystals have excellent PM ability in both the visible and UV regions on the basis of Kurtz and Perry’s rule^51^ (Figure 4d–g). As shown in Figure 4h, for the incident radiation at 1064 nm, in the identical maximum particle size range, the SHG responses for K_2_B_4_SO_10_, Rb_2_B_4_SO_10_, Cs_2_B_4_SO_10_, and Rb_3_B_11_PO_19_F_3_ are 0.9, 0.9, 1.7, and 1.3 × KDP, respectively. The relative magnitudes of their SHG responses under 532 nm incident radiation were 0.1, 0.1, 0.3, and 0.2 × β-BBO, respectively (Figure 4i). The SHG effect of this series of compounds is essentially at the same level as that of KBBF (1.2 × KDP). The results also show that the SHG capacities of the A_2_B_4_SO_10_ (A = NH_4_, K, Rb, and Cs) and A_3_B_11_PO_19_F_3_ (M = NH_4_ and Rb) families are in the identical order of magnitude.^8,44^

### Thermal Stability Test

To assess thermal stability, the TG-DSC curves of these four compounds were tested (Figure S3). The weight losses of K_2_B_4_SO_10_, Rb_2_B_4_SO_10_, and Cs_2_B_4_SO_10_ start at 606, 609, and 614 °C, and the corresponding endothermic peaks occur at 660, 757, and 753 °C on the DSC curves during the measurement, respectively (Figure S3a–c). The weight losses are about 20% below 800 °C, which may be associated with the decomposition of borosulfates by releasing SO_2_ gas. These measurement results suggest that K_2_B_4_SO_10_, Rb_2_B_4_SO_10_, and Cs_2_B_4_SO_10_ display good thermal stability up to 600 °C. The weight decrease of Rb_3_B_11_PO_19_F_3_ starts at 470 °C (Figure S3d) and is not significant (∼3% up to 800 °C), corresponding to the endothermic peak at 544 °C. Based on other literature reports,^38^ the weight reduction may be a result of the decomposition of Rb_3_B_11_PO_19_F_3_ by releasing BF_3_ gas. It can be concluded that Rb_3_B_11_PO_19_F_3_ can be stable up to 470 °C.

### Infrared Spectroscopy

The infrared spectra of these four compounds were measured, which led to the identification of their anionic groups. The infrared peak positions for K_2_B_4_SO_10_, Rb_2_B_4_SO_10_, and Cs_2_B_4_SO_10_ do not differ much (Figure S4a). According to the literature studies,^45,47^ the asymmetric and symmetric stretching vibrations (ν_asym_ and ν_sym_) of the [BO_3_] units are observed at 1363–1375 and 944–950 cm^–1^, respectively. The vibrational peaks of 803–813 and 582–584 cm^–1^ are caused by the out-of-plane bending [δ(O–B–O)] of the [BO_3_] groups. The ν_sym_ of the [BO_4_] tetrahedra are between 1105 and 1115 cm^–1^. The very strong band around 1221–1224 cm^–1^ can be ascribed to the ν_asym_ of the [SO_4_] units. The ν_sym_ of the [SO_4_] units are around 508–512 cm^–1^. For Rb_3_B_11_PO_19_F_3_ (Figure S4b), the ν_asym_ and ν_sym_ of the [BO_3_] groups are detected at 1423 as well as 899 cm^–1^, respectively. Meanwhile, the δ(O–B–O) of the [BO_3_] units is recorded at 698 and 577 cm^–1^. The bands centered at 1233, 1087, 850, and 609 cm^–1^ are ascribed to the ν_asym_ and ν_sym_ of the tetrahedral groups [BO_3_F], [BO_4_], and [PO_4_], respectively. In particular, the peak around 770 cm^–1^ is caused by the stretching vibrations of the B–F bonds, which proves the presence of the fluorine atoms in the Rb_3_B_11_PO_19_F_3_. These results probe the coordination modes of the anionic units of K_2_B_4_SO_10_, Rb_2_B_4_SO_10_, Cs_2_B_4_SO_10_, and Rb_3_B_11_PO_19_F_3_. At the same time, the energy-dispersive X-ray spectra prove the existence of B, S, O, and K/Rb/Cs elements in K_2_B_4_SO_10_/Rb_2_B_4_SO_10_/Cs_2_B_4_SO_10_, and B, P, O, F, and Rb elements in Rb_3_B_11_PO_19_F_3_ (Figure S5). The above-mentioned test results further validate the plausibility of these four compounds.

### Numerical Calculation Details

The calculated band structures of K_2_B_4_SO_10_, Rb_2_B_4_SO_10_, Cs_2_B_4_SO_10_, and Rb_3_B_11_PO_19_F_3_ indicate that they have band gaps of 5.00, 5.08, 5.29, and 5.91 eV (Figure S6), respectively. The total density of states (DOS) curves for K_2_B_4_SO_10_, Rb_2_B_4_SO_10_, and Cs_2_B_4_SO_10_ are quite similar (Figure S7). From the partial densities of states (PDOS), we can clearly find that the upper regions of valence bands (VBs) are chiefly made of the O 2p orbitals. The bottom of conduction bands (CBs) is dominantly formed by B 2p, S 3p, and O 2p orbitals, suggesting that [BO_3_], [BO_4_], and [SO_4_] units contribute to the band gaps of K_2_B_4_SO_10_, Rb_2_B_4_SO_10_, and Cs_2_B_4_SO_10_. For Rb_3_B_11_PO_19_F_3_ (Figure S7), the B 2p, P 3p, O 2p, and F 2p orbitals constitute the maximum region surrounding VBs and the minimum region of CBs, implying that the [BO_3_], [BO_4_], [BO_3_F], and [PO_4_] units determine the band gap. It follows that the electronic transition near the forbidden bands in K_2_B_4_SO_10_, Rb_2_B_4_SO_10_, Cs_2_B_4_SO_10_, and Rb_3_B_11_PO_19_F_3_ is mainly determined by the functional [B_4_SO_10_]_∞_ and [B_5_PO_10_F]_∞_ layers, respectively.

**Figure 5:**
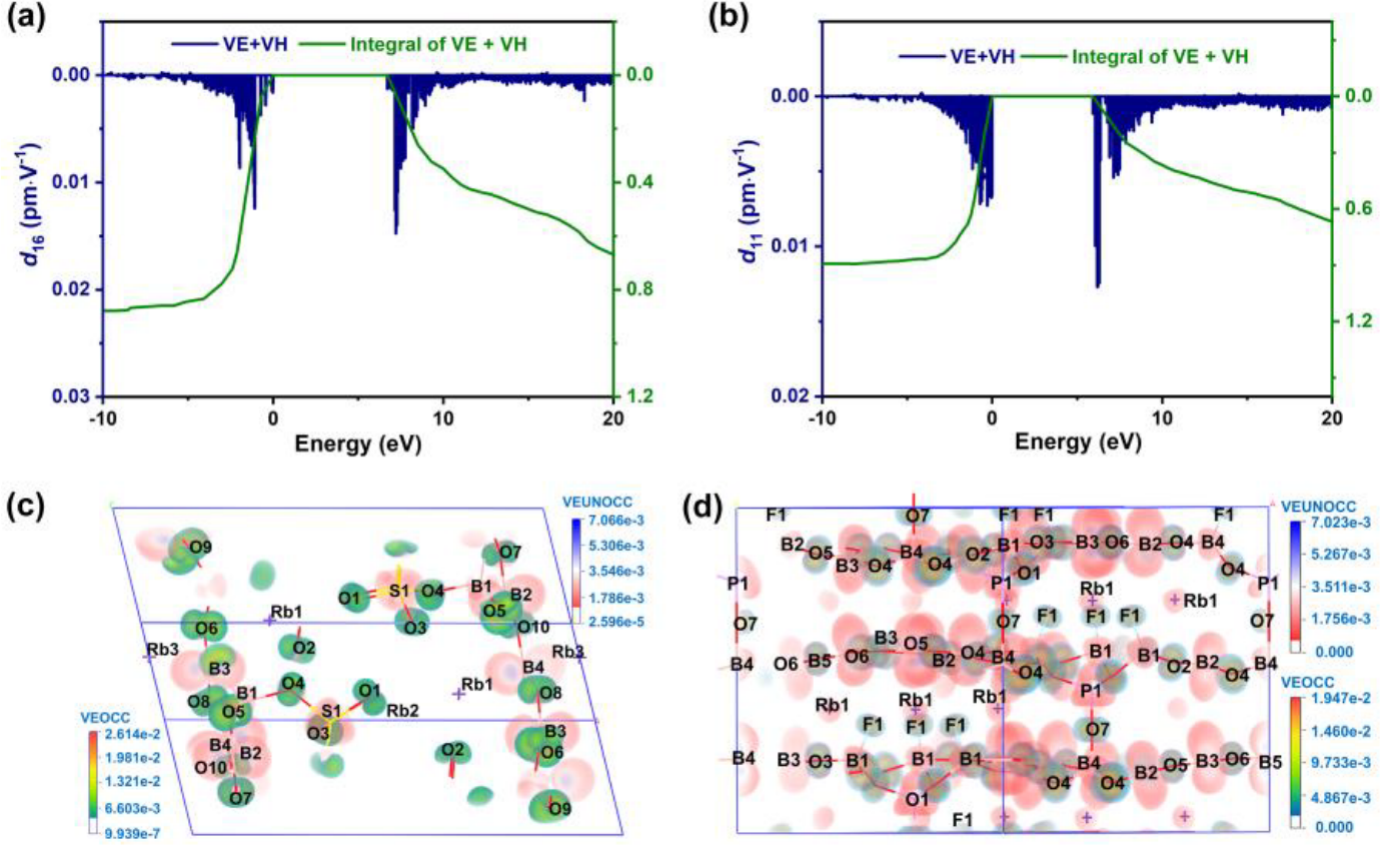
Band-resolved analysis of Rb_2_B_4_SO_10_ (a) and Rb_3_B_11_PO_19_F_3_ (b). SHG density maps of occupied and unoccupied orbitals of the largest NLO tensor *d*_16_ for Rb_2_B_4_SO_10_ (c) and *d*_11_ for Rb_3_B_11_PO_19_F_3_ (d) in a virtual-electron process.

K_2_B_4_SO_10_, Rb_2_B_4_SO_10_, and Cs_2_B_4_SO_10_ all belong to point group 2; hence, within the limitation of Kleinman symmetry, each of them possesses four independent nonzero NLO coefficients, i.*e*., *d*_14_, *d*_16_, *d*_22_, and *d*_23_ (Table S8).^52,53^ In the principal planes, the effective nonlinearity (*d*_eff_) expressions exist with three tensors *d*_14_, *d*_16_, and *d*_23_, in which the largest tensor *d*_16_ for K_2_B_4_SO_10_, Rb_2_B_4_SO_10_, and Cs_2_B_4_SO_10_ is 1.7–2.3 × KDP (*d*_36_ = 0.39 · pm V^–1^). For Rb_3_B_11_PO_19_F_3_, that crystallizes in point group 3, which possesses four independent nonzero NLO coefficients *d*_11_, *d*_31_, *d*_22_, and *d*_33_. The *d*_eff_ expressions include *d*_11_, *d*_31_, and *d*_22_, of which the largest tensor *d*_11_ is 2.8 × KDP (Table S8). The calculated analyses for these four compounds are in the identical order of magnitude as the measured powder SHG effect (0.9–1.7 × KDP). To prove the provenience of NLO performance, the band-resolved analysis^54^ was performed based on the largest coefficient *d*_16_ and *d*_11_ for K_2_B_4_SO_10_, Rb_2_B_4_SO_10_, Cs_2_B_4_SO_10_, and Rb_3_B_11_PO_19_F_3_, respectively. Generally, electronic exchanges in the neighborhood of the Fermi level (FL) determine the optical properties of these four compounds. For K_2_B_4_SO_10_, Rb_2_B_4_SO_10_, and Cs_2_B_4_SO_10_, the SHG effect predominantly results from the contribution of orbitals in the section of the VBs and the bottom part of the CBs, *i*.e., the electronic states of B, S, and O atoms that belong to the [B_4_SO_10_]_∞_ layer (Figure 5a and Figure S8). For Rb_3_B_11_PO_19_F_3_, p orbitals of B, P, O, and F atoms near the FL are dominantly responsible for the SHG process, which indicates the decisive role of the [B_5_PO_10_F]_∞_ layer (Figure 5b). Meanwhile, SHG-weighted electron density analysis was adopted to further visualize the source of the SHG effect.^55^ The SHG process contains the virtual electron (VE) process and virtual hole (VH) process, in which the VE process makes dominant contributions (94.6%, 92.1%, 91.0%, and 93.1%) for K_2_B_4_SO_10_, Rb_2_B_4_SO_10_, Cs_2_B_4_SO_10_, and Rb_3_B_11_PO_19_F_3_, respectively. Therefore, the occupied and unoccupied states for these four compounds in the VE process were visualized. Based on the SHG-weighted density map of *d*_16_ for K_2_B_4_SO_10_, Rb_2_B_4_SO_10_, and Cs_2_B_4_SO_10_, it is evidently found that strong SHG responses stem from the interaction between [BO_3_], [BO_4_], and [SO_4_] units (Figure 5c and Figure S8). For Rb_3_B_11_PO_19_F_3_, it can be concluded that the donation of the SHG responses to *d*_11_ primarily stems from the [BO_3_] units and slightly from [BO_3_F], [BO_4_], and [PO_4_] units (Figure 5d). These results are consistent with those of the band-resolved analysis.

**Figure 6:**
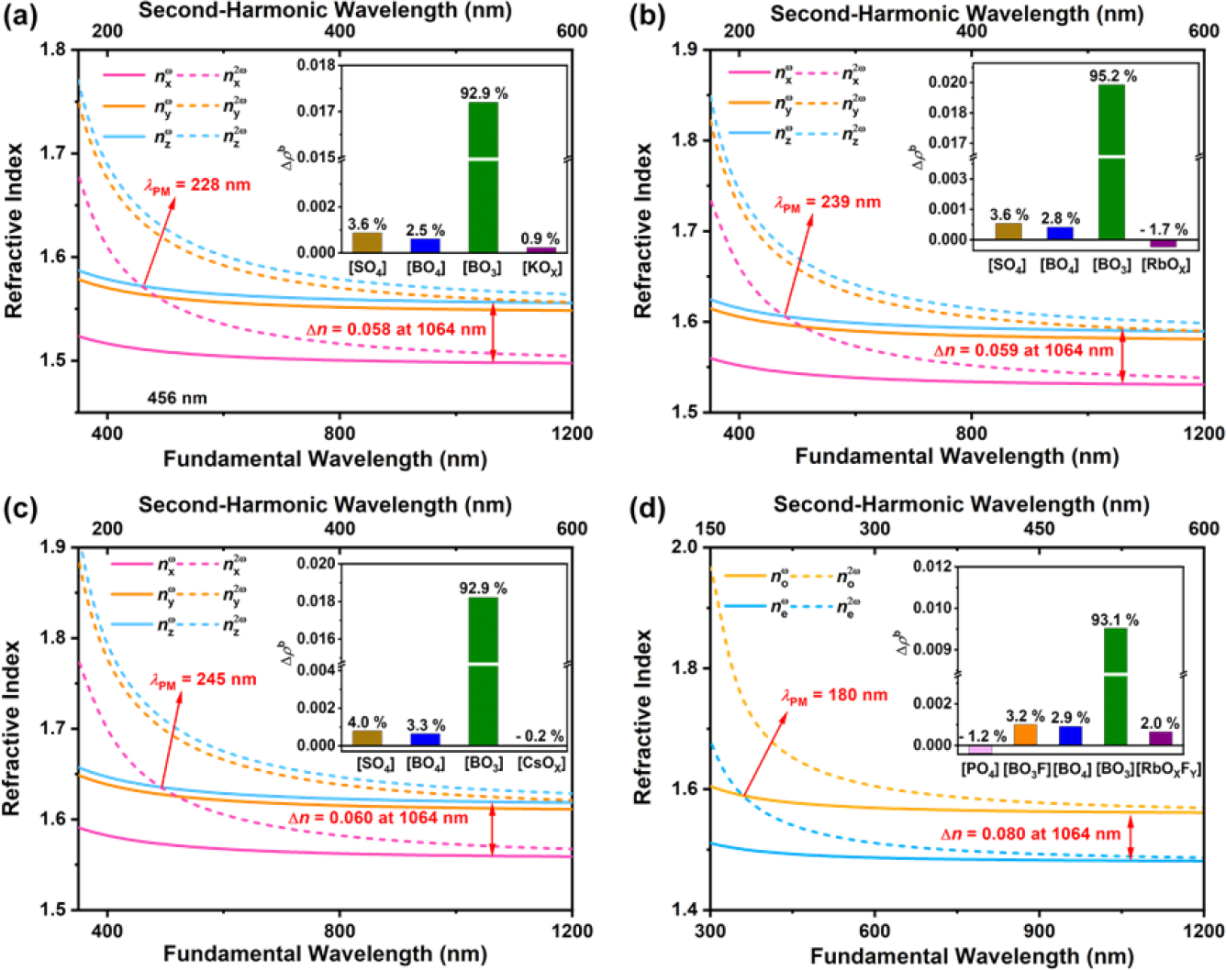
Calculated results of bonding electron density difference (Δρ^b^) in the upper-right corner of the diagram and refractive indices of K_2_B_4_SO_10_ (a), Rb_2_B_4_SO_10_ (b), Cs_2_B_4_SO_10_ (c), and Rb_3_B_11_PO_19_F_3_ (d).

Based on the first-principle-calculated refractive index dispersion curves in Figure 6, the calculated birefringences are 0.058, 0.059, 0.060, and 0.080 at 1064 nm for K_2_B_4_SO_10_, Rb_2_B_4_SO_10_, Cs_2_B_4_SO_10_, and Rb_3_B_11_PO_19_F_3_, respectively. According to the REDA analysis^56^ (as shown in the insets of Figure 6), the contributions of [BO_3_] groups in the [B_4_SO_10_]_∞_ layers for K_2_B_4_SO_10_, Rb_2_B_4_SO_10_, and Cs_2_B_4_SO_10_ are about 92.9%, 95.2%, and 92.9%, respectively, and that in the [B_11_PO_19_F_3_]_∞_ layer for Rb_3_B_11_PO_19_F_3_ is about 93.1%, while other groups also contribute slightly to the birefringence. Extrapolating from the above calculation results, the large birefringence of K_2_B_4_SO_10_, Rb_2_B_4_SO_10_, Cs_2_B_4_SO_10_, and Rb_3_B_11_PO_19_F_3_ is strongly related to the almost coplanar arrangement of the large optically anisotropic [BO_3_] groups in the KBBF-type [B_4_SO_10_]_∞_ and [B_11_PO_19_F_3_]_∞_ layers. The shortest λ_PM_ values of K_2_B_4_SO_10_, Rb_2_B_4_SO_10_, Cs_2_B_4_SO_10_, and Rb_3_B_11_PO_19_F_3_ are determined based on the condition of the type-I PM process, which can reach about 228, 239, 245, and 180 nm, respectively (Figure 6).^57^ The λ_PM_ values of K_2_B_4_SO_10_, Rb_2_B_4_SO_10_, and Cs_2_B_4_SO_10_ are all less than 266 nm which indicates that they can produce the FHG of the 1064 nm Nd:YAG laser. Better still, the shortest λ_PM_ of Rb_3_B_11_PO_19_F_3_ is about 180 nm, which implies that Rb_3_B_11_PO_19_F_3_ breaks the limitation of λ_PM_ less than 200 nm.

## Conclusion

In summary, new borosulfates M_2_B_4_SO_10_ (M = K, Rb, and Cs) and fluoroborophosphate Rb_3_B_11_PO_19_F_3_ crystals were triumphantly designed and synthesized derived from the classical KBBF structure. Since M_2_B_4_SO_10_ and Rb_3_B_11_PO_19_F_3_ inherit the beneficial layer structure of KBBF, they show a remarkable linear and nonlinear optical performance including short UV cutoff edges (<190 nm), large SHG effect (0.9–1.7 × KDP), and moderate birefringence (Δ*n* = 0.058–0.080 at 1064 nm) to realize the output short-wavelength UV laser light through SHG. Remarkably, the calculated shortest λ_PM_ of Rb_3_B_11_PO_19_F_3_ is about 180 nm, which means that the Rb_3_B_11_PO_19_F_3_ crystal is a powerful competitor as a deep-UV NLO material. In addition to this, first-principles calculations certify that the KBBF-type [B_4_SO_10_]_∞_ and [B_11_PO_19_F_3_]_∞_ layers are the source of their excellent optical properties. This work demonstrates that it is effective to probe short-wavelength UV NLO crystals in mixed-anion systems by using the KBBF template.

## Supporting Information Available

Details of experimental and calculation methods; bond lengths and angles; UV–vis–NIR spectra; crystal photographs; TG-DSC analyses; IR spectra; EDS spectra; first-principle calculations; and powder XRD patterns (PDF)
CCDC-2093133 for K_2_B_4_SO_10_ (CIF)
CCDC-2093131 for Rb_2_B_4_SO_10_ (CIF)
CCDC-2093132 for Cs_2_B_4_SO_10_ (CIF)
CCDC-20178806 for Rb_3_B_11_PO_19_F_3_ (CIF)

## Supplementary Material

oc2c00832_si_001.pdf

oc2c00832_si_002.cif

oc2c00832_si_003.cif

oc2c00832_si_004.cif

oc2c00832_si_005.cif
