# Supplementary material for: Exploring Short-Wavelength Phase-Matching Nonlinear Optical Crystals by Employing KBe2BO3F2 as the Template
Source: ACS Cent Sci. 2022 Nov 11;8(11):1557–64. doi: 10.1021/acscentsci.2c00832 (PMC9686211; doi:10.1021/acscentsci.2c00832)
Supplement: Supplementary file 1 — oc2c00832_si_001.pdf [file oc2c00832_si_001.pdf]

# Exploring Short-Wavelength Phase-Matching Nonlinear Optical Crystals by Employing $KBe_2BO_3F_2$ as the Template

Zijian Li,[a,b] Wenqi Jin,[a,b] Fangfang Zhang,[a,b,*] Zhihua Yang,[a,b] Shilie Pan[a,b,*]

[a]*Research Center for Crystal Materials, CAS Key Laboratory of Functional Materials and Devices for Special Environments, Xinjiang Technical Institute of Physics and Chemistry of CAS, 40-1 South Beijing Road, Urumqi 830011, China*

[b]*Center of Materials Science and Optoelectronics Engineering, University of Chinese Academy of Sciences, Beijing 100049, China*

[*]*To whom correspondence should be addressed, E-mails: ffzhang@ms.xjb.ac.cn (Fangfang Zhang), slpan@ms.xjb.ac.cn (Shilie Pan)*

## EXPERIMENTAL SECTION

**1. Polycrystalline Preparation and Crystal Growth** The starting agents of $K_2SO_4$ (99.99 %), $Rb_2SO_4$ (99.0 %), $Cs_2SO_4$ (99.9 %), $Rb_2CO_3$ (99.9 %), $HPF_6$ (~ 60 wt. %), and $B_2O_3$ (98 %) were acquired from Aladdin Chemical Industry Co., Ltd., and used without additional treatment. Polycrystalline $M_2B_4SO_{10}$ were synthesized *via* the traditional solid-state reaction method by using stoichiometric ratios of $M_2SO_4$ and $B_2O_3$ based on the equation $M_2SO_4 + 2B_2O_3 = M_2B_4SO_{10}$. Stoichiometric proportions of $M_2SO_4$ and $B_2O_3$ were mixed, ground, and transferred into quartz tubes that were sealed under vacuum (about $10^{-3}$ Pa). Afterward, the tubes were heated from 40 to 450 °C in 10 h, dwell at this temperature for 72 h, and subsequently lowered to 40 °C with a cooling rate of 10 $°C \cdot h^{-1}$. After the reaction, solid powders of $M_2B_4SO_{10}$ were obtained. For $Rb_3B_{11}PO_{19}F_3$, the $RbPF_6$ precursor was synthesized by reaction of $Rb_2CO_3$ and the excess amount of $HPF_6$. After filtration, white powders of $RbPF_6$ were obtained and further dried at 120 °C. Traditional solid-state sintering method can be used to obtain polycrystalline $Rb_3B_{11}PO_{19}F_3$ based on the following equation $RbPF_6 + Rb_2CO_3 + 6B_2O_3 = Rb_3B_{11}PO_{19}F_3 + BF_3$. Stoichiometric proportions of $RbPF_6$, $Rb_2CO_3$, and $B_2O_3$ were ground thoroughly and placed into a quartz tube that was fame sealed under vacuum (about $10^{-3}$ Pa). The sample was steadily heated to 350 ºC in 10 h and dwelled at this temperature for 100 h before being cooled to 200 ºC in 150 h, and after the reaction, solid chunks of $Rb_3B_{11}PO_{19}F_3$ were obtained.

The high-temperature solution growth approach was adopted to grow single crystals of $M_2B_4SO_{10}$ based on the following different temperature profiles in a closed system. The starting reactants of $M_2SO_4$ (M = K, Rb, and Cs) and $B_2O_3$ were weighed in the molar ratio 1: 4 (with excess $B_2O_3$ as the flux) and placed into evacuated quartz tubes after thorough mixing. Then the tubes were heated for 10 h from 40 to 500 °C for $K_2B_4SO_{10}$, and 600 °C for $Rb_2B_4SO_{10}$ and $Cs_2B_4SO_{10}$, respectively, dwelled at these temperatures for 48 h followed by cooling to the normal atmospheric temperature at a rate of 1 $°C \cdot h^{-1}$. The single crystal of $Rb_3B_{11}PO_{19}F_3$ was obtained in the same way as $M_2B_4SO_{10}$. Reagents of $RbPF_6$, $Rb_2CO_3$, and $B_2O_3$ were mixed thoroughly in the molar ratio of 1: 1: 4, and transferred into a quartz tube, besides, the tube was flame-sealed under vacuum (about $10^{-3}$ Pa). The sample was slowly heated to 450 ºC in 10 h and dwelled at this temperature for 48 h before cooling to 200 ºC in 150 h. Dozens of colorless sheet-like crystals with high yield were obtained.

**2. Characterization.** Transparent sub-millimeter-sized single crystals of $M_2B_4SO_{10}$ and $Rb_3B_{11}PO_{19}F_3$ were selected under polarization contrast microscopy for single-crystal X-ray diffraction (XRD) measurements. Diffraction data of $M_2B_4SO_{10}$ and $Rb_3B_{11}PO_{19}F_3$ were gathered by a Bruker D8 Venture diffractometer (at 300 K) equipped with Mo Kα radiation ($\lambda$ = 0.71073 Å) and Cu Kα radiation ($\lambda$ = 1.54056 Å), respectively. Intensity corrections for Lorentz and polarization effects were carried out in the SAINT program.[1] The structure resolution and parameter refinement of these four compounds (full-matrix least-squares against $F^2$) were carried out by using the SHELX-2014 suite nested on the olex2 software.[2] Since these $M_2B_4SO_{10}$ and $Rb_3B_{11}PO_{19}F_3$ crystallize in the chiral Sohncke space group,[3] other enantiomers can be calculated through structure inversion by using the SHELX-14 suite on olex2

software. Any missed symmetry elements were checked with PLATON.[4] In $Rb_3B_{11}PO_{19}F3$, the O7 atoms in the B-O-P linkage are off the triad axis (C3), which results in its structure disordered. The crystallographic parameters for the four structures were briefly listed in Table S1. The atomic position final refinement parameters, selected bond distances, and angles are provided in Table S2-7, respectively. Powder XRD tests for all four compounds were performed on Bruker D2 Advance X-ray diffractometer using monochromated Cu Kα radiation (λ = 1.5418 Å). The powder XRD patterns for $M_2B_4SO_{10}$ and $Rb_3B_{11}PO_{19}F_3$ are shown in Figure S9, and it demonstrates that the experimental profiles are consistent with the theoretical ones. Microprobe elemental analyses were conducted via a field emission scanning electron microscope (Hitachi S-4800, Japan) with an energy-dispersive X-ray spectroscope (EDS). The thermal behaviors of $M_2B_4SO_{10}$ and $Rb_3B_{11}PO_{19}F_3$ were studied with thermogravimetry and differential scanning calorimeter (TG-DSC) by using a NETZSCH STA 449F3 simultaneous thermal instrument. The measurements were carried out in flowing nitrogen at a heating rate of 5 °C·min$^{-1}$ from 40 to 800 °C. Infrared (IR) spectra were recorded on a Shimadzu IR Affinity-1 IR spectrometer ranging from 4000 to 500 cm$^{-1}$. UV-vis-NIR diffuse reflectance spectra were tested with a Shimadzu SolidSpec-3700 DUV spectrophotometer in the wavelength range of 200-2600 nm. Transmittance spectra from 180 to 1600 nm were recorded by using a UV-Vis-NIR spectrophotometer (Cary 5000, Agilent Technologies) based on unpolished crystal wafers with the thicknesses of 0.3 mm. The visible and UV SHG performance was measured with the Kurtz-Perry method[5] with laser wavelengths at 1064 and 532 nm, respectively, which were radiated from a Q-switched Nd:YVO4 laser. Before the measurements, polycrystalline samples of $M_2B_4SO_{10}$ and $Rb_3B_{11}PO_{19}F_3$ were gently ground into a fine powder and sieved into six different particle size ranges (38-55, 55-88, 88-105, 105-150, 150-200, and 200-250 μm). Microcrystalline KDP and *β*-BBO, which are also ground into the identical particle size range as mentioned above, were regarded as a standard for visible and UV SHG measurements, respectively.

**3. Computational Details.** Electronic structure calculations were made with the CASTEP software using the density functional theory (DFT).[6] During the calculation, the generalized gradient approximation (GGA) with Perdew-Burke-Ernzerhof (PBE) functional was adopted.[7] The plane-wave energy cutoff was set at 750 eV, and the separation of the k-point was set as 0.04 Å–1 in the Brillouin zone. Since exchange-correlation energy in the DFT is discontinuous, the calculations will underestimate the band gaps.[8,9] For $M_2B_4SO_{10}$, we utilized scissor operators to shift the conduction bands upward to approach experimental values. For $Rb_3B_{11}PO_{19}F_3$, the difference between the HSE06 and GGA band gaps was chosen as the scissors operator value. The NLO coefficients were computed by employing the so-called length-gauge formalism derived by Aversa and Sipe at zero frequency.[10–13] To inspect the source of the SHG response, the band-resolved method and SHG-weighted density analysis were carried out.[14,15] To compute the birefringence, the complex dielectric function $\varepsilon(\omega) = \varepsilon_1(\omega) + i\varepsilon_2(\omega)$ was calculated in the arbitrary phase approximation from the PBE wave functions. The dielectric constant's real part was acquired by a

Kramers-Kronig transformation of the calculated imaginary part, from which the refractive indices were deduced.[16] The response electron distribution anisotropy (REDA) method was adopted to elucidate the origin of birefringence, which was developed to identify the correlation between optical anisotropy and the distribution of bonding electrons in compounds.[17]

**Table 1.** Crystal Data and Structure Refinements for $M_2B_4SO_{10}$ and $Rb_3B_{11}PO_{19}F_3$.

| Formula | $K_2B_4SO_{10}$ | $Rb_2B_4SO_{10}$ | $Cs_2B_4SO_{10}$ | $Rb_3B_{11}PO_{19}F_3$ |
|---|---|---|---|---|
| Formula weight | 313.50 | 406.24 | 501.12 | 767.29 |
| Temperature / K | | 300.0 | | |
| Crystal system | | monoclinic | | trigonal |
| Space group | | *C*2 | | *R*3 |
| $a$ / Å | 11.2631(9) | 11.3127(8) | 11.4012(6) | 11.37150(10) |
| $b$ / Å | 6.4339(4) | 6.5152(5) | 6.5997(4) | 11.37150(10) |
| $c$ / Å | 12.6486(11) | 12.9709(11) | 13.5702(7) | 12.0240(3) |
| $\alpha$ / ° | 90 | 90 | 90 | 90 |
| $\beta$ / ° | 105.707(4) | 105.411(3) | 103.934(2) | 90 |
| $\gamma$ / ° | 90 | 90 | 90 | 120 |
| Volume / Å$^3$ | 882.36(12) | 921.64(12) | 991.04(9) | 1346.53(4) |
| $Z$ | | 4 | | 3 |
| $\rho_{calc}$ / g·cm$^{-3}$ | 2.360 | 2.928 | 3.359 | 2.839 |
| Abs coeff. / mm$^{-1}$ | 1.352 | 10.886 | 7.606 | 12.478 |
| $F$ (000) | 616.0 | 760.0 | 904.0 | 1080.0 |
| Crystal size / mm$^3$ | 0.099 × 0.073 × 0.048 | 0.131 × 0.062 × 0.041 | 0.161 × 0.074 × 0.049 | 0.122 × 0.08 × 0.073 |
| Radiation | | Mo Kα ($\lambda$ = 0.71073 Å) | | Cu Kα ($\lambda$ = 1.54178 Å) |
| 2$\theta$ range / ° | 6.692 to 55.054 | 3.26 to 55.12 | 6.186 to 54.93 | 11.614 to 136.482 |
| Index ranges | $-14 \le h \le 14, -8 \le k \le 8, -16 \le l \le 16$ | $-13 \le h \le 14, -8 \le k \le 8, -16 \le l \le 16$ | $-12 \le h \le 14, -8 \le k \le 8, -17 \le l \le 17$ | $-13 \le h \le 13, -11 \le k \le 13, -14 \le l \le 14$ |
| Reflns collected | 10380 | 8729 | 8724 | 5714 |
| Unique reflns ($R_{int}$) | 2032 [$R_{int}$ = 0.0727] | 2120 [$R_{int}$ = 0.0622] | 2274 [$R_{int}$ = 0.0426] | 1085 [$R_{int}$ = 0.0287] |
| Data / restraints / param | 2032 / 1 / 156 | 2120 / 19 / 155 | 2274 / 1 / 156 | 1085/1/119 |
| GOF on $F^2$ | 1.103 | 1.055 | 1.069 | 1.080 |

| | | | | |
|---|---|---|---|---|
| $R_1$[a]/ $wR_2$[b] [I ≥ 2σ(I)] | 0.0462 / 0.1068 | 0.0346 / 0.0634 | 0.0241 / 0.0464 | 0.0277 / 0.0774 |
| $R_1$[a]/ $wR_2$[b] [all data] | 0.0599 / 0.1168 | 0.0447 / 0.0677 | 0.0272 / 0.0483 | 0.0279 / 0.0778 |
| Residual electron density (min/max) / e·Å$^{-3}$ | 0.44 / – 0.56 | 0.76 / – 0.98 | 0.56 / – 0.80 | 0.79 / – 0.53 |
| Flack parameter | – 0.02(12) | 0.012(11) | 0.028(19) | 0.06(4) |

[a]$R_1 = \Sigma||F_o| - |F|| / \Sigma|F_o|$. [b]$wR_2 = [\Sigma w(F_o^2 - F_c^2)^2/\Sigma wF_o^4]^{1/2}$ for $F_o^2 > 2\sigma(F_o^2)$

**Table S2.** Fractional atomic coordinates (×$10^4$) and equivalent isotropic displacement parameters (Å$^2$×$10^3$) for $M_2B_4SO_{10}$ (M = K, Rb, and Cs). $U_{eq}$ is defined as 1/3 of the trace of the orthogonalised $U_{ij}$ tensor.

| **Atom** | **x** | **y** | **z** | $U_{(eq)}$ |
|---|---|---|---|---|
| | | $K_2B_4SO_{10}$ | | |
| K1 | 1073.3(15) | 4757(4) | 2986.1(13) | 54.7(5) |
| K2 | 5000 | 4985(6) | 5000 | 64.6(8) |
| K3 | 5000 | 9703(3) | 10000 | 29.9(4) |
| S1 | 2023.3(15) | 4709(3) | 6139.3(13) | 39.4(4) |
| B1 | 1825(5) | 3222(9) | 8095(6) | 19.7(13) |
| B2 | 3958(6) | 4228(9) | 8696(6) | 21.7(13) |
| B3 | 6120(5) | 4797(10) | 8666(5) | 19.5(11) |
| B4 | 7426(5) | 1747(10) | 8682(6) | 20.9(13) |
| O1 | 1500(6) | 4008(12) | 5047(5) | 64.5(18) |
| O2 | 3353(5) | 4461(14) | 6489(5) | 67(2) |
| O3 | 1467(4) | 3184(7) | 6848(4) | 32.3(10) |
| O4 | 1674(7) | 6786(9) | 6356(6) | 64.3(19) |
| O5 | 3124(3) | 2701(6) | 8522(4) | 22.4(9) |
| O6 | 3650(3) | 6260(6) | 8814(4) | 27.0(10) |
| O7 | 5169(3) | 3657(6) | 8840(4) | 23.8(9) |
| O8 | 5993(3) | 6806(6) | 8413(4) | 27.6(10) |
| O9 | 7232(3) | 3818(6) | 8793(4) | 28.4(10) |
| O10 | 6548(3) | 287(6) | 8470(3) | 20.5(8) |
| | | $Rb_2B_4SO_{10}$ | | |
| Rb1 | 3903.8(5) | 2870.1(13) | 6977.2(4) | 31.63(14) |
| Rb2 | 5000 | -2267(2) | 5000 | 37.1(2) |
| Rb3 | 5000 | 2824.9(14) | 0 | 19.17(14) |
| S1 | 2958.1(12) | 3019(3) | 3805.1(10) | 24.2(3) |
| B1 | 3140(5) | 4349(9) | 1847(6) | 15.3(12) |
| B2 | 2583(5) | 10798(9) | 1377(6) | 17.2(13) |
| B3 | 3862(4) | 7725(12) | 1336(4) | 12.5(9) |
| B4 | 6025(5) | 8291(7) | 1305(5) | 14.1(12) |
| O1 | 3440(5) | 3853(7) | 4860(4) | 52.9(14) |
| O2 | 1628(4) | 3155(9) | 3443(3) | 42.2(13) |
| O3 | 3390(5) | 957(7) | 3671(4) | 46.7(14) |
| O4 | 3478(3) | 4497(5) | 3078(3) | 20.8(9) |
| O5 | 3955(3) | 5724(5) | 1528(3) | 15.6(7) |
| O6 | 2785(3) | 8757(5) | 1287(3) | 22.7(9) |
| O7 | 3433(3) | 12285(4) | 1548(3) | 14.4(8) |
| O8 | 4822(3) | 8854(5) | 1162(3) | 16.8(8) |
| O9 | 6352(3) | 6278(5) | 1246(3) | 18.8(9) |
| O10 | 6849(3) | 9828(5) | 1429(3) | 12.6(8) |
| | | $Cs_2B_4SO_{10}$ | | |
| Cs1 | 3895.9(4) | 4113.5(12) | 6887.5(3) | 26.50(13) |

| | | | | |
|---|---|---|---|---|
| Cs2 | 5000 | -933(2) | 5000 | 33.06(17) |
| Cs3 | 5000 | 4025.7(13) | 0 | 18.65(14) |
| S1 | 2917.0(17) | 4336(4) | 3740.3(13) | 23.2(5) |
| B1 | 3095(8) | 5566(11) | 1849(7) | 13.8(15) |
| B2 | 3857(6) | 8930(18) | 1369(5) | 15.2(13) |
| B3 | 6029(7) | 9462(11) | 1352(6) | 17.6(18) |
| B4 | 7574(8) | 7038(11) | 1418(6) | 16.1(16) |
| O1 | 3481(7) | 2386(10) | 3704(6) | 57(2) |
| O2 | 3221(8) | 5350(12) | 4702(5) | 71(3) |
| O3 | 1621(5) | 4151(18) | 3354(4) | 43.9(15) |
| O4 | 3392(5) | 5793(7) | 3013(4) | 21.3(11) |
| O5 | 3907(4) | 6935(7) | 1527(4) | 18.0(10) |
| O6 | 4838(4) | 9990(6) | 1214(4) | 19.5(11) |
| O7 | 6832(5) | 10986(7) | 1452(4) | 14.5(11) |
| O8 | 6369(5) | 7469(8) | 1288(5) | 19.6(12) |
| O9 | 8421(4) | 8500(6) | 1601(4) | 15.5(10) |
| O10 | 7824(4) | 5013(6) | 1354(4) | 20.7(11) |

$U_{eq}$ is defined as one-third of the trace of the orthogonalized $U_{ij}$ tensor.

**Table S3.** Selected bond lengths (Å) and angles (deg.) for $K_2B_4SO_{10}$.

| | | | |
|---|---|---|---|
| K1-O1 | 2.565(6) | K3-O8$^{10\#}$ | 3.157(5) |
| K1-O2$^{2\#}$ | 3.129(9) | K3-O8 | 3.157(5) |
| K1-O3$^{3\#}$ | 3.095(5) | K3-O9$^{11\#}$ | 3.130(4) |
| K1-O4$^{4\#}$ | 3.105(7) | K3-O9$^{12\#}$ | 3.130(4) |
| K1-O5$^{2\#}$ | 2.996(5) | K3-O10$^{9\#}$ | 2.958(4) |
| K1-O6$^{4\#}$ | 3.275(5) | K3-O10$^{8\#}$ | 2.958(4) |
| K1-O8$^{4\#}$ | 3.150(4) | S1-O1 | 1.421(6) |
| K1-O9$^{5\#}$ | 3.375(5) | S1-O2 | 1.451(5) |
| K1-O10$^{2\#}$ | 3.047(4) | S1-O3 | 1.569(5) |
| K2-O1$^{6\#}$ | 3.082(8) | S1-O4 | 1.439(7) |
| K2-O1$^{2\#}$ | 3.082(8) | B1-O3 | 1.518(9) |
| K2-O2 | 2.999(5) | B1-O5 | 1.455(7) |
| K2-O2$^{5\#}$ | 2.999(5) | B2-O5 | 1.336(7) |
| K2-O3$^{6\#}$ | 3.216(5) | B2-O6 | 1.371(7) |
| K2-O3$^{2\#}$ | 3.216(5) | B2-O7 | 1.376(7) |
| K2-O4$^{7\#}$ | 2.997(7) | B3-O7 | 1.365(7) |
| K2-O4$^{4\#}$ | 2.997(7) | B3-O8 | 1.330(8) |
| K3-O5$^{8\#}$ | 3.089(4) | B3-O9 | 1.372(7) |
| K3-O5$^{9\#}$ | 3.089(4) | B4-O9 | 1.364(7) |
| K3-O6 | 2.870(4) | B4-O10 | 1.337(7) |
| K3-O6$^{10\#}$ | 2.870(4) | O5$^{8\#}$-K3-O5$^{9\#}$ | 102.72(16) |
| K3-O7$^{9\#}$ | 2.968(4) | O5$^{9\#}$-K3-O9$^{11\#}$ | 52.45(10) |
| K3-O7$^{8\#}$ | 2.968(4) | O5$^{8\#}$-K3-O8$^{10\#}$ | 106.60(12) |
| O1-K1-O2$^{2\#}$ | 89.7(2) | O6-K3-O5$^{8\#}$ | 167.77(11) |
| O1-K1-O4$^{4\#}$ | 72.4(2) | O6$^{10\#}$-K3-O5$^{9\#}$ | 167.77(11) |
| O1-K1-O3$^{3\#}$ | 77.99(18) | O6$^{10\#}$-K3-O5$^{8\#}$ | 89.24(12) |
| O1-K1-O9$^{5\#}$ | 131.27(17) | O6$^{10\#}$-K3-O10$^{9\#}$ | 96.72(11) |
| O2$^{2\#}$-K1-O9$^{5\#}$ | 101.12(14) | O7$^{8\#}$-K3-O9$^{12\#}$ | 95.93(11) |
| O3$^{3\#}$-K1-O8$^{4\#}$ | 44.37(12) | O8-K3-O8$^{10\#}$ | 107.64(15) |
| O3$^{3\#}$-K1-O9$^{5\#}$ | 133.36(13) | O9$^{11\#}$-K3-O9$^{12\#}$ | 159.04(16) |
| O4$^{4\#}$-K1-O9$^{5\#}$ | 60.01(14) | O10$^{9\#}$-K3-O5$^{8\#}$ | 83.63(11) |
| O5$^{2\#}$-K1-O9$^{5\#}$ | 50.68(10) | O10$^{9\#}$-K3-O9$^{11\#}$ | 112.92(12) |
| O5$^{2\#}$-K1-O4$^{4\#}$ | 101.22(14) | O10$^{8\#}$-K3-O10$^{9\#}$ | 165.42(16) |
| O5$^{2\#}$-K1-O10$^{2\#}$ | 87.38(11) | O1-S1-O2 | 112.6(4) |
| O1$^{2\#}$-K2-O3$^{7\#}$ | 69.37(16) | O1-S1-O3 | 103.4(4) |
| O2$^{5\#}$-K2-O1$^{7\#}$ | 71.2(2) | O4-S1-O2 | 110.9(5) |
| O2-K2-O3$^{2\#}$ | 104.18(16) | O5-B2-O6 | 122.1(5) |
| O2$^{5\#}$-K2-O3$^{7\#}$ | 104.18(16) | O7-B3-O9 | 117.8(5) |
| O2$^{5\#}$-K2-O3$^{2\#}$ | 84.19(17) | O10-B4-O6$^{7\#}$ | 121.7(5) |
| O3$^{6\#}$-K2-O3$^{2\#}$ | 100.4(2) | O1-S1-O4 | 114.6(4) |
| O4$^{7\#}$-K2-O1$^{2\#}$ | 147.61(18) | O4-S1-O3 | 107.4(3) |
| O4$^{4\#}$-K2-O4$^{7\#}$ | 93.2(3) | O5-B2-O7 | 116.9(5) |
| O4$^{4\#}$-K2-O1$^{2\#}$ | 107.99(18) | O8-B3-O7 | 121.5(5) |

| | | | |
|---|---|---|---|
| O5$^{9\#}$-K3-O9$^{11\#}$ | 52.45(10) | O10-B4-O9 | 125.2(5) |

Symmetry transformations used to generate equivalent atoms:

$^{1\#}$ – 1/2 + X, – 1/2 + Y, – 1 + Z; $^{2\#}$ 1/2 – X, 1/2 + Y, 1 – Z; $^{3\#}$ – X, + Y, 1 – Z; $^{4\#}$ 1/2 – X, – 1/2 + Y, 1 – Z;

$^{5\#}$ 1 – X, + Y, 1 – Z; $^{6\#}$ 1/2 + X, 1/2 + Y, + Z; $^{7\#}$ 1/2 + X, – 1/2 + Y, + Z; $^{8\#}$ 1 – X, 1 + Y, 2 – Z;

$^{9\#}$ + X, 1 + Y, + Z; $^{10\#}$ 1 – X, + Y, 2 – Z; $^{11\#}$ – 1/2 + X, 1/2 + Y, + Z; $^{12\#}$ 3/2 – X, 1/2 + Y, 2 – Z;

**Table S4.** Selected bond lengths (Å) and angles (deg.) for $Rb_2B_4SO_{10}$.

| | | | |
|---|---|---|---|
| Rb1-O1 | 2.732(5) | Rb3-O7$^{9\#}$ | 3.032(3) |
| Rb1-O2$^{1\#}$ | 3.150(6) | Rb3-O7$^{5\#}$ | 3.032(3) |
| Rb1-O2$^{2\#}$ | 3.513(6) | Rb3-O8$^{5\#}$ | 3.028(3) |
| Rb1-O3$^{2\#}$ | 3.214(5) | Rb3-O8$^{9\#}$ | 3.028(3) |
| Rb1-O4$^{3\#}$ | 3.165(4) | Rb3-O9 | 2.949(4) |
| Rb1-O4$^{1\#}$ | 3.463(4) | Rb3-O9$^{7\#}$ | 2.949(4) |
| Rb1-O5$^{3\#}$ | 3.254(3) | Rb3-O10$^{5\#}$ | 3.092(4) |
| Rb1-O6$^{1\#}$ | 3.367(4) | Rb3-O10$^{9\#}$ | 3.092(4) |
| Rb1-O7$^{4\#}$ | 3.134(3) | Rb2$^{10\#}$-O1 | 3.061(5) |
| Rb1-O9$^{3\#}$ | 3.268(4) | Rb2$^{11\#}$-O2 | 1.371(7) |
| Rb1-O10$^{4\#}$ | 3.141(4) | Rb1$^{12\#}$-O7 | 3.134(3) |
| Rb2-O1$^{5\#}$ | 3.061(5) | S1-O1 | 1.438(5) |
| Rb2-O1$^{4\#}$ | 3.061(5) | S1-O2 | 1.455(4) |
| Rb2-O2$^{6\#}$ | 3.086(4) | S1-O3 | 1.456(5) |
| Rb2-O2$^{1\#}$ | 3.087(4) | S1-O4 | 1.567(4) |
| Rb2-O3 | 3.006(5) | B1-O4 | 1.544(8) |
| Rb2-O3$^{3\#}$ | 3.006(5) | B1-O5 | 1.423(7) |
| Rb2-O4$^{4\#}$ | 3.368(4) | B2-O6 | 1.359(6) |
| Rb2-O4$^{5\#}$ | 3.368(4) | B2-O7 | 1.341(6) |
| Rb3-O5 | 3.185(4) | B3-O5 | 1.326(8) |
| Rb3-O5$^{7\#}$ | 3.185(4) | B3-O6 | 1.378(6) |
| Rb3-O6$^{6\#}$ | 3.207(4) | B3-O8 | 1.379(6) |
| Rb3-O6$^{8\#}$ | 3.207(4) | B4-O8 | 1.373(6) |
| O1-Rb1-O2$^{1\#}$ | 94.47(13) | B4-O9 | 1.371(6) |
| O1-Rb-O2$^{2\#}$ | 68.61(13) | B4-O10 | 1.348(6) |
| O1-Rb1-O3$^{2\#}$ | 70.39(15) | O6$^{8\#}$-Rb3-O6$^{6\#}$ | 158.17(12) |
| O1-Rb1-O4$^{3\#}$ | 80.19(13) | O5$^{7\#}$-Rb3-O6$^{6\#}$ | 74.68(9) |
| O1-Rb1-O10$^{4\#}$ | 142.80(12) | O7$^{9\#}$-Rb3-O10$^{9\#}$ | 87.21(9) |
| O2$^{1\#}$-Rb1-O3$^{2\#}$ | 117.47(11) | O7$^{9\#}$-Rb3-O10$^{5\#}$ | 84.39(9) |
| O3$^{2\#}$-Rb1-O5$^{3\#}$ | 102.70(10) | O9$^{7\#}$-Rb3-O5$^{7\#}$ | 55.32(9) |
| O4$^{3\#}$-Rb1-O2$^{2\#}$ | 77.94(9) | O1-S1-O2 | 112.5(3) |
| O7$^{4\#}$-Rb1-O2$^{2\#}$ | 108.13(9) | O2-S1-O4 | 106.8(3) |
| O9$^{3\#}$-Rb1-O2$^{2\#}$ | 53.12(9) | O3-S1-O4 | 107.7(3) |
| O9$^{3\#}$-Rb1-B1$^{3\#}$ | 75.74(13) | O5-B1-O4 | 105.0(4) |
| O10$^{4\#}$-Rb1-O2$^{1\#}$ | 54.94(10) | O5-B1-O10$^{14\#}$ | 114.9(5) |
| O10$^{4\#}$-Rb1-O4$^{1\#}$ | 42.92(9) | O5-B1-O4 | 105.0(4) |
| O10$^{4\#}$-Rb1-O5$^{3\#}$ | 104.92(9) | O10$^{14\#}$-B1-O7$^{5\#}$ | 112.1(4) |
| O10$^{4\#}$-Rb1-O9$^{3\#}$ | 82.76(9) | O7-B2-O6 | 126.3(5) |
| O2$^{6\#}$-Rb2-O4$^{5\#}$ | 81.27(12) | O7-B2-O9$^{11\#}$ | 120.5(5) |
| O3$^{3\#}$-Rb2-O1$^{5\#}$ | 149.69(13) | O5-B3-O8 | 121.9(4) |
| O3$^{3\#}$-Rb2-O2$^{6\#}$ | 86.40(14) | O5-B3-O6 | 120.8(4) |
| O3-Rb2-O4$^{4\#}$ | 167.12(11) | O9-B4-O8 | 121.4(4) |
| O5$^{7\#}$-Rb3-O5 | 107.24(12) | O10-B4-O9 | 122.0(5) |

Symmetry transformations used to generate equivalent atoms:

1# 1/2 – X, – 1/2 + Y, 1 – Z; 2# 1/2 – X, 1/2 + Y, 1 – Z; 3# 1 – X, + Y, 1 – Z; 4# 1 – X, – 1 + Y, 1 – Z;
5# + X, – 1 + Y, + Z; 6# 1/2 + X, – 1/2 + Y, + Z; 7# 1 – X, + Y, – Z; 8# 1/2 – X, – 1/2 + Y, – Z;
9# 1 – X, – 1 + Y, – Z; 10# + X, 1 + Y, + Z; 11# – 1/2 + X, 1/2 + Y, + Z; 12# 1 – X, 1 + Y, 1 – Z;
13# 1/2 + X, 1/2 + Y, + Z; 14# – 1/2 + X, – 1/2 + Y, + Z

**Table S5.** Selected bond lengths (Å) and angles (deg.) for $Cs_2B_4SO_{10}$.

| | | | |
|---|---|---|---|
| Cs1-O1$^{1\#}$ | 2.732(5) | Cs3-O6$^{9\#}$ | 3.207(4) |
| Cs1-O1$^{2\#}$ | 3.150(6) | Cs3-O7$^{6\#}$ | 3.032(3) |
| Cs1-O2 | 2.992(7) | Cs3-O7$^{9\#}$ | 3.032(3) |
| Cs1-O3$^{3\#}$ | 3.330(12)- | Cs3-O8$^{8\#}$ | 3.028(3) |
| Cs1-O3$^{2\#}$ | 3.165(4) | Cs3-O8 | 3.028(3) |
| Cs1-O4$^{3\#}$ | 3.434(5) | Cs3-O9$^{10\#}$ | 3.157(5) |
| Cs1-O4$^{1\#}$ | 3.257(5) | Cs3-O9$^{11\#}$ | 3.157(5) |
| Cs1-O5$^{1\#}$ | 3.431(5) | Cs3-O10 | 3.364(5) |
| Cs1-O7$^{4\#}$ | 3.134(3) | Cs3-O10$^{8\#}$ | 3.364(5) |
| Cs1-O8$^{1\#}$ | 3.268(4) | S1-O1 | 1.444(7) |
| Cs1-O9$^{5\#}$ | 3.272(5) | S1-O2 | 1.433(7) |
| Cs1-O10$^{1\#}$ | 3.061(5) | S1-O3 | 1.449(5) |
| Cs2-O1 | 3.069(7) | S1-O4 | 1.565(5) |
| Cs2-O1$^{1\#}$ | 3.069(7) | B1-O4 | 1.541(10) |
| Cs2-O2$^{4\#}$ | 3.146(7) | B1-O5 | 1.435(9) |
| Cs2-O2$^{6\#}$ | 3.146(7) | B2-O5 | 1.333(13) |
| Cs2-O3$^{7\#}$ | 3.224(5) | B2-O6 | 1.378(9) |
| Cs2-O3$^{3\#}$ | 3.368(4) | B3-O6 | 1.370(9) |
| Cs2-O4$^{4\#}$ | 3.595(5) | B3-O7 | 1.345(9) |
| Cs2-O4$^{6\#}$ | 3.185(4) | B3-O8 | 1.380(9) |
| Cs3-O5 | 3.282(5) | B4-O8 | 1.372(9) |
| Cs3-O5$^{8\#}$ | 3.207(4) | B4-O9 | 1.345(9) |
| O1$^{1\#}$-Cs1-O1$^{2\#}$ | 146.6(3) | O5$^{8\#}$-Cs3-O10$^{8\#}$ | 90.03(12) |
| O1$^{1\#}$-Cs1-O4$^{3\#}$ | 81.11(13) | O6$^{6\#}$-Cs3O7$^{9\#}$ | 71.46(14)- |
| O1$^{1\#}$-Cs1-O5$^{2\#}$ | 70.39(15) | O6$^{9\#}$-Cs3-O10 | 106.24(12) |
| O2-Cs1-O7$^{4\#}$ | 143.03(17) | O6$^{9\#}$-Cs3-O10$^{8\#}$ | 92.68(12) |
| O2-Cs1-O8$^{2\#}$ | 119.93(19) | O8-Cs3-O9$^{10\#}$ | 100.26(13) |
| O2-Cs1-O9$^{5\#}$ | 129.30(19) | O8$^{8\#}$-Cs3-O9$^{11\#}$ | 100.26(13) |
| O3$^{1\#}$-Cs1-O10$^{2\#}$ | 77.59(11) | O8-Cs3-O10$^{8\#}$ | 117.27(13) |
| O4$^{3\#}$-Cs1-O1$^{2\#}$ | 120.29(14) | O1-S1-O3 | 109.7(6) |
| O5$^{2\#}$-Cs-O4$^{3\#}$ | 140.19(12) | O1-S1-O4 | 107.5(4) |
| O7$^{4\#}$-Cs1-O1$^{2\#}$ | 108.94(16) | O2-S1-O3 | 111.7(5) |
| O8$^{2\#}$-Cs1-O1$^{1\#}$ | 63.40(16) | O10$^{12\#}$-B2-O6 | 117.3(9) |
| O9$^{5\#}$-Cs1-O1$^{2\#}$ | 51.45(16) | O5-B2-O6 | 121.4(7) |
| O1$^{2\#}$-Cs2-O2$^{6\#}$ | 152.53(18) | O6-B3-O8 | 121.2(6) |
| O1-Cs2-O3$^{7\#}$ | 86.0(2) | O7-B3-O6 | 116.8(6) |
| O2$^{6\#}$-Cs2-O3$^{7\#}$ | 112.6(2) | O7-B3-O8 | 121.6(6) |
| O2$^{6\#}$-Cs2-O4$^{6\#}$ | 40.39(13) | O8-B4-Cs3 | 52.9(4) |
| O3$^{7\#}$-Cs2-O4$^{6\#}$ | 76.38(17) | O9-B-Cs3 | 158.5(5) |
| O5$^{8\#}$-Cs3-O10 | 76.87(12) | O9-B4-O8 | 121.8(6) |
| O8-Cs3-O10$^{8\#}$ | 117.27(13) | O9-B4-O10 | 124.0(7) |

Symmetry transformations used to generate equivalent atoms:

$^{1\#}$ 1/2 – X, 1/2 + Y, 1 – Z; $^{2\#}$ 1 – X, + Y, 1 – Z; $^{3\#}$ 1/2 – X, -1/2 + Y, 1 – Z; $^{4\#}$ 1 – X, – 1 + Y, 1 – Z;

[5#] 3/2 – X, -1/2 + Y, 1 – Z; [6#] + X, – 1 + Y ,+ Z; [7#] 1/2 + X, -1/2 + Y, + Z; [8#] 1 – X, + Y, – Z;

[9#] 1 – X, – 1 + Y, - Z; [10#] 3/2 – X, – 1/2 + Y, - Z; [11#] – 1/2 + X, – 1/2 + Y, + Z; [12#] – 1/2 + X, 1/2 + Y, + Z;

**Table S6.** Fractional Atomic Coordinates (×10[4]) and Equivalent Isotropic Displacement Parameters ($Å^2×10^3$) for $Rb_3B_{11}PO_{19}F_3$. $U_{eq}$ is defined as 1/3 of the trace of the orthogonalised $U_{IJ}$ tensor.

| **Atom** | **x** | **y** | **z** | **$^aU_{(eq)}$** | **Occupancy** |
|---|---|---|---|---|---|
| Rb1 | -3339.5(7) | 103.1(6) | -7070.4(13) | 29.1(3) | 1 |
| P1 | -3333.33 | -6666.67 | -4417(3) | 20.2(6) | 1 |
| B1 | -3592(8) | -4715(8) | -5540(7) | 24.3(17) | 1 |
| B2 | -1996(8) | -2326(9) | -5219(8) | 30(2) | 1 |
| B3 | -4352(8) | -3077(7) | -5476(7) | 19.3(14) | 1 |
| B4 | 0 | 0 | -5319(10) | 18(2) | 1 |
| B5 | -6666.67 | -3333.33 | -5560(9) | 17(2) | 1 |
| O1 | -4003(5) | -5880(6) | -4770(7) | 56(2) | 1 |
| O2 | -2291(6) | -3647(6) | -5224(6) | 35.1(15) | 1 |
| O3 | -4663(5) | -4376(5) | -5517(5) | 25.8(12) | 1 |
| O4 | -704(6) | -1379(6) | -5012(7) | 49.2(19) | 1 |
| O5 | -3023(6) | -2025(5) | -5370(5) | 26.7(12) | 1 |
| O6 | -5280(5) | -2642(5) | -5578(5) | 27.6(11) | 1 |
| O7 | -332(14) | 283(14) | -6562(9) | 28(3) | 0.333333 |
| F1 | -3527(6) | -5158(7) | -6602(5) | 71.7(19) | 1 |

$U_{eq}$ is defined as one-third of the trace of the orthogonalized $U_{ij}$ tensor.

**Table S7.** Selected bond lengths (Å) and angles (deg.) for $Rb_3B_{11}PO_{19}F_3$.

| | | | |
|---|---|---|---|
| Rb1-F1$^{1\#}$ | 3.037(5) | P1-O7$^{10\#}$ | 1.552(11) |
| Rb1-O1$^{2\#}$ | 3.001(6) | B1-F1 | 1.388(10) |
| Rb1-O2$^{3\#}$ | 3.254(6) | B1-O1 | 1.488(10) |
| Rb1-O2$^{4\#}$ | 3.128(6) | B1-O2 | 1.418(10) |
| Rb1-O3$^{1\#}$ | 3.047(6) | B1-O3 | 1.450(10) |
| Rb1-O3$^{2\#}$ | 3.210(5) | B2-O2 | 1.366(10) |
| Rb1-O4$^{4\#}$ | 3.188(6) | B2-O4 | 1.341(10) |
| Rb1-O5 | 3.322(5) | B2-O5 | 1.383(10) |
| Rb1-O5$^{5\#}$ | 3.137(5) | B3-O3 | 1.337(9) |
| Rb1-O6$^{5\#}$ | 3.399(5) | B3-O5 | 1.387(10) |
| Rb1-O6 | 3.308(5) | B3-O6 | 1.377(9) |
| Rb1-O7 | 3.378(13) | B4-O4 | 1.407(7) |
| P1-O1$^{6\#}$ | 1.497(5) | B4-O7 | 1.614(15) |
| P1-O1$^{7\#}$ | 1.497(5) | B5-O6 | 1.366(5) |
| P1-O1 | 1.497(5) | | |
| F1$^{1\#}$-Rb1-O2$^{2\#}$ | 148.20(18) | O3$^{1\#}$-Rb1-O6$^{5\#}$ | 92.68(13) |
| F1$^{1\#}$-Rb1-O2$^{3\#}$ | 79.01(17) | O3$^{4\#}$-Rb1-O6$^{5\#}$ | 51.84(13) |
| F1$^{1\#}$-Rb1-O3$^{4\#}$ | 93.81(15) | O3$^{1\#}$-Rb1-O6 | 53.98(14) |
| F1$^{1\#}$-Rb1-O3$^{1\#}$ | 44.53(15) | O3$^{4\#}$-Rb1-O7 | 76.5(2) |
| F1$^{1\#}$-Rb1-O4$^{2\#}$ | 109.88(16) | O3$^{1\#}$-Rb1-O7 | 131.4(2) |
| F1$^{1\#}$-Rb1-O5 | 126.82(15) | O4$^{2\#}$-Rb1-O2$^{3\#}$ | 162.87(17) |
| F1$^{1\#}$-Rb1-O5$^{5\#}$ | 61.81(17) | O4$^{2\#}$-Rb1-O3$^{4\#}$ | 108.5(2) |
| F1$^{1\#}$-Rb1-O6 | 97.96(14) | O4$^{2\#}$-Rb1-O5 | 81.58(17) |
| F1$^{1\#}$-Rb1-O6$^{5\#}$ | 53.04(14) | O4$^{2\#}$-Rb1-O6$^{5\#}$ | 91.92(17) |
| F1$^{1\#}$-Rb1-O7 | 140.7(3) | O4$^{2\#}$-Rb1-O6 | 61.58(19) |
| O1$^{4\#}$-Rb1-F3$^{1\#}$ | 135.59(15) | O4$^{2\#}$-Rb1-O7 | 109.3(2) |
| O1$^{4\#}$-Rb1-O2$^{3\#}$ | 81.93(19) | O5$^{5\#}$-Rb1-O2$^{3\#}$ | 138.74(10) |
| O1$^{4\#}$-Rb-O2$^{2\#}$ | 58.27(16) | O5$^{5\#}$-Rb1-O3$^{4\#}$ | 85.38(14) |
| O1$^{4\#}$-Rb1-O3$^{4\#}$ | 44.55(16) | O5$^{5\#}$-Rb1-O4$^{2\#}$ | 55.44(14) |
| O1$^{4\#}$-Rb1-O3$^{1\#}$ | 165.94(19) | O5$^{5\#}$-Rb1-O5 | 131.82(4) |
| O1$^{4\#}$-Rb1-O4$^{2\#}$ | 99.8(2) | O5$^{5\#}$-Rb1-O6 | 94.77(15) |
| O1$^{4\#}$-Rb1-O5 | 88.82(17) | O5-Rb1-O6$^{5\#}$ | 172.98(13) |
| O1$^{4\#}$-Rb1-O5$^{5\#}$ | 116.7(2) | O5$^{5\#}$-Rb1-O6$^{5\#}$ | 41.17(13) |
| O1$^{4\#}$-Rb1-O6 | 125.46(13) | O5-Rb1-O7 | 57.2(3) |
| O1$^{4\#}$-Rb1-O6$^{5\#}$ | 94.94(16) | O5$^{5\#}$-Rb1-O7 | 151.1(2) |
| O1$^{4\#}$-Rb-O7 | 36.5(2) | O6-Rb1-O5 | 40.80(13) |
| O2$^{2\#}$-Rb1-O2$^{3\#}$ | 132.05(6) | O6-Rb1-O6$^{5\#}$ | 133.20(4) |
| O2$^{2\#}$-Rb1-O3$^{4\#}$ | 84.15(15) | O6-Rb1-O7 | 97.9(2) |
| O2$^{2\#}$-Rb1-O4$^{2\#}$ | 42.82(16) | O7-Rb1-O6$^{5\#}$ | 128.2(2) |
| O2$^{3\#}$-Rb1-O5 | 81.42(14) | O7$^{8\#}$-P1-O7$^{7\#}$ | 39.5(8) |
| O2$^{2\#}$-Rb1-O5 | 72.86(15) | O7$^{6\#}$-P1-O7$^{7\#}$ | 39.5(8) |
| O2$^{2\#}$-Rb1-O5$^{5\#}$ | 86.42(15) | O7$^{6\#}$-P1-O7$^{8\#}$ | 39.5(8) |
| O2$^{2\#}$-Rb1-O6 | 82.62(14) | O1$^{10\#}$-P1-O1$^{9\#}$ | 112.3(3) |

| | | | |
|---|---|---|---|
| O2$^{2\#}$-Rb1-O6$^{5\#}$ | 104.12(16) | O1$^{10\#}$-P1-O1 | 112.3(3) |
| O2$^{3\#}$-Rb1-O65 | 104.95(13) | O1$^{9\#}$-P1-O1 | 112.3(3) |
| O2$^{3\#}$-Rb1-O6 | 103.40(16) | O1$^{10\#}$-P1-O7$^{8\#}$ | 83.6(6) |
| O2$^{3\#}$-Rb1-O7 | 62.4(2) | O1$^{9\#}$-P1-O7$^{7\#}$ | 114.7(6) |
| O2$^{2\#}$-Rb1-O7 | 69.6(2) | O1-P1-O7$^{8\#}$ | 114.7(6) |
| O3$^{1\#}$-Rb1-O2$^{2\#}$ | 130.80(11) | O1$^{9\#}$-P1-O7$^{8\#}$ | 118.5(6) |
| O3$^{1\#}$-Rb1-O2$^{3\#}$ | 84.70(14) | F1-B1-O1 | 107.4(7) |
| O3$^{4\#}$-Rb1-O2$^{3\#}$ | 84.81(15) | F1-B1-O2 | 109.6(7) |
| O3$^{1\#}$-Rb1-O3$^{4\#}$ | 138.28(5) | F1-B1-O3 | 108.6(7) |
| O3$^{1\#}$-Rb1-O4$^{2\#}$ | 91.69(18) | O2-B1-O1 | 108.9(7) |
| O3$^{4\#}$-Rb1-O5 | 132.87(10) | O2-B1-O3 | 115.0(6) |
| O3$^{1\#}$-Rb1-O5 | 84.91(14) | O2-B2-O5 | 119.9(7) |
| O3$^{1\#}$-Rb1-O5$^{5\#}$ | 76.57(13) | O3-B1-O1 | 107.1(6) |
| O3$^{4\#}$-Rb1-O6 | 166.73(13) | O3-B3-O5 | 122.0(7) |
| O3-B3-O6 | 124.5(7) | O4-B4-O7$^{12\#}$ | 83.2(6) |
| O4-B2-O2 | 116.8(6) | O4$^{12\#}$-B4-O7$^{12\#}$ | 113.9(8) |
| O4-B2-O5 | 123.1(7) | O4$^{3\#}$-B4-O7 | 83.2(6) |
| O4-B4-O4$^{12\#}$ | 113.4(4) | O4-B4-O7$^{3\#}$ | 116.2(8) |
| O4-B4-O4$^{3\#}$ | 113.4(5) | O6$^{1\#}$-B5-O6 | 119.98(3) |
| O4$^{12\#}$-B4-O4$^{3\#}$ | 113.4(5) | O6$^{1\#}$-B5-O61$^{1\#}$ | 119.97(3) |
| O4$^{12\#}$-B4-O7 | 116.2(8) | O6-B5-O61$^{1\#}$ | 119.97(3) |
| O4$^{3\#}$-B4-O7$^{12\#}$ | 116.2(8) | O6-B3-O5 | 113.5(6) |
| O4-B4-O7 | 113.9(8) | O7-B4-O7$^{3\#}$ | 38.0(8) |
| O4$^{3\#}$-B4-O7$^{3\#}$ | 113.9(8) | O7-B4-O7$^{12\#}$ | 38.0(8) |
| O4$^{12\#}$-B4-O7$^{3\#}$ | 83.2(6) | O7$^{12\#}$-B4-O7$^{3\#}$ | 38.0(8) |

Symmetry codes for $Rb_3B_{11}PO_{19}F_3$:

$^{1\#}$ – 1 – Y, + X – Y, – Z; $^{2\#}$ – 2/3 – Y, – 1/3 + X – Y, – 1/3 + Z; $^{3\#}$ + Y – X, – X, + Z;

$^{4\#}$ 1/3 + X, 2/3 + Y, – 1/3 + Z; $^{5\#}$ – 2/3 + Y – X, – 1/3 – X, – 1/3 + Z; $^{6\#}$ 1/3 – Y, – 2/3 + X – Y, 1/3 + Z;

$^{7\#}$ – 1/3 + X, – 2/3 + Y, 1/3 + Z; $^{8\#}$ – 1/3 + Y – X, – 2/3 – X,1/3 + Z; $^{9\#}$ + Y – X, – 1 – X, + Z;

$^{10\#}$ – 1 – Y, – 1 + X – Y, + Z; $^{11\#}$ – 1 + Y – X, – 1 – X, + Z; $^{12\#}$ – Y, + X – Y, + Z;

**Table S8.** The calculated SHG coefficients of $M_2B_4SO_{10}$ (M = K, Rb, and Cs) and $Rb_3B_{11}PO_{19}F_3$.

| Compound | SHG coefficients (pm·V$^{-1}$) | | | |
|---|---|---|---|---|
| | $d_{16} = d_{21}$ | $d_{14} = d_{25} = d_{36}$ | $d_{23} = d_{34}$ | $d_{22}$ |
| $K_2B_4SO_{10}$ | – 0.831 | 0.058 | – 0.298 | 1.069 |
| $Rb_2B_4SO_{10}$ | 0.872 | 0.059 | 0.450 | – 1.221 |
| $Cs_2B_4SO_{10}$ | 0.665 | 0.060 | 0.551 | – 1.084 |
| $Rb_3B_{11}PO_{19}F_3$ | $d_{11} = d_{12} = -d_{26}$ | $d_{15} = d_{24} = d_{31} = d_{32}$ | $d_{22} = -d_{16} = d_{21}$ | $d_{33}$ |
| | 1.077 | 0.002 | 0.897 | 0.122 |

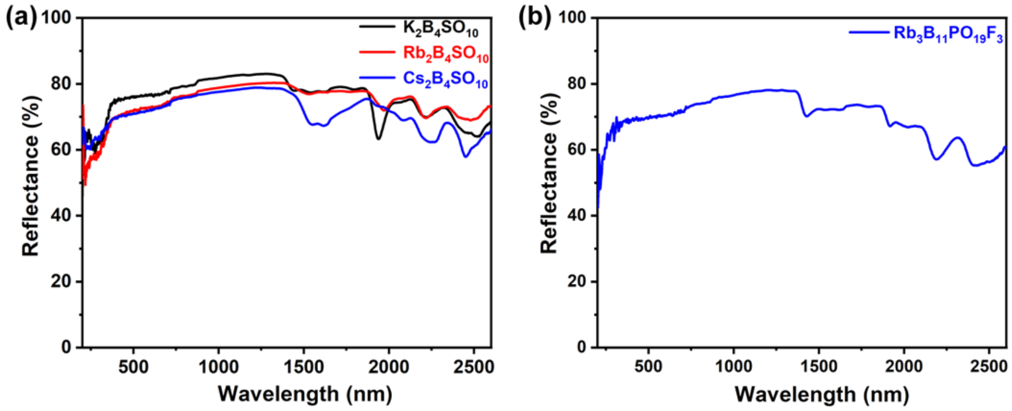


**Figure S1.** Optical diffuse reflectance spectra of $M_2B_4SO_{10}$ (M = K, Rb, and Cs) (a) and $Rb_3B_{11}PO_{19}F_3$ (b).

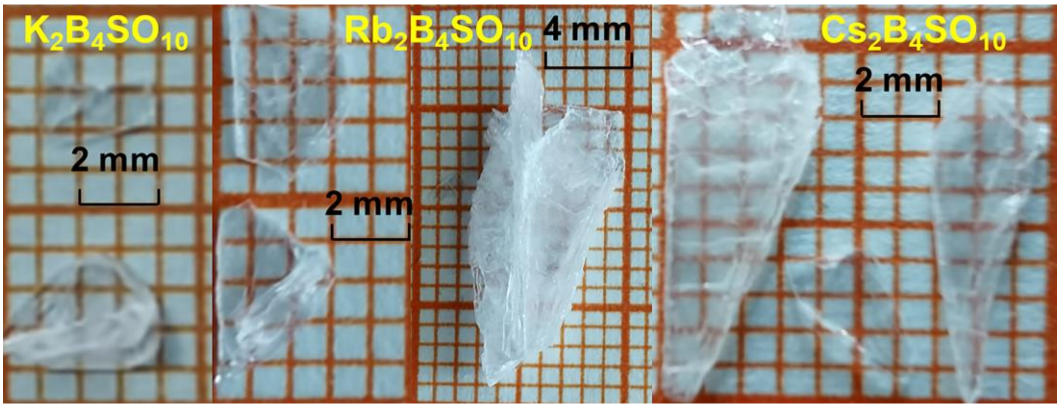

**Figure S2.** Photographs of $K_2B_4SO_{10}$, $Rb_2B_4SO_{10}$, and $Cs_2B_4SO_{10}$ crystals.

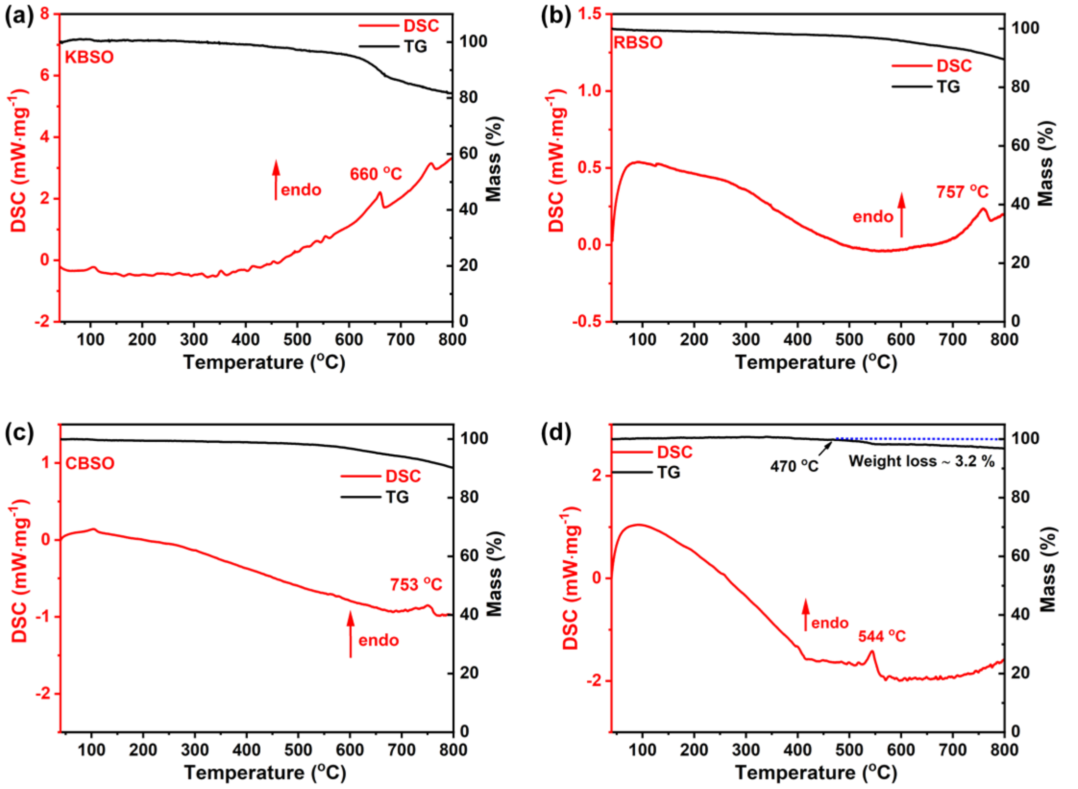


**Figure S3.** Thermogravimetric (TG) and differential scanning calorimetry (DSC) curves of $K_2B_4SO_{10}$ (a), $Rb_2B_4SO_{10}$ (b), $Cs_2B_4SO_{10}$ (c), and $Rb_3B_{11}PO_{19}F_3$ (d).

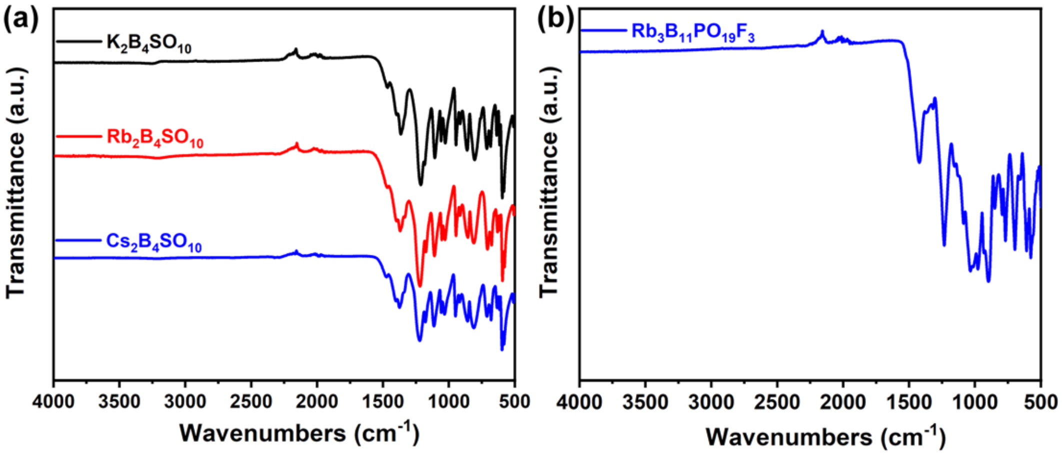


**Figure S4.** IR spectra of $M_2B_4SO_{10}$ (M = K, Rb, and Cs) (a) and $Rb_3B_{11}PO_{19}F_3$ (b).

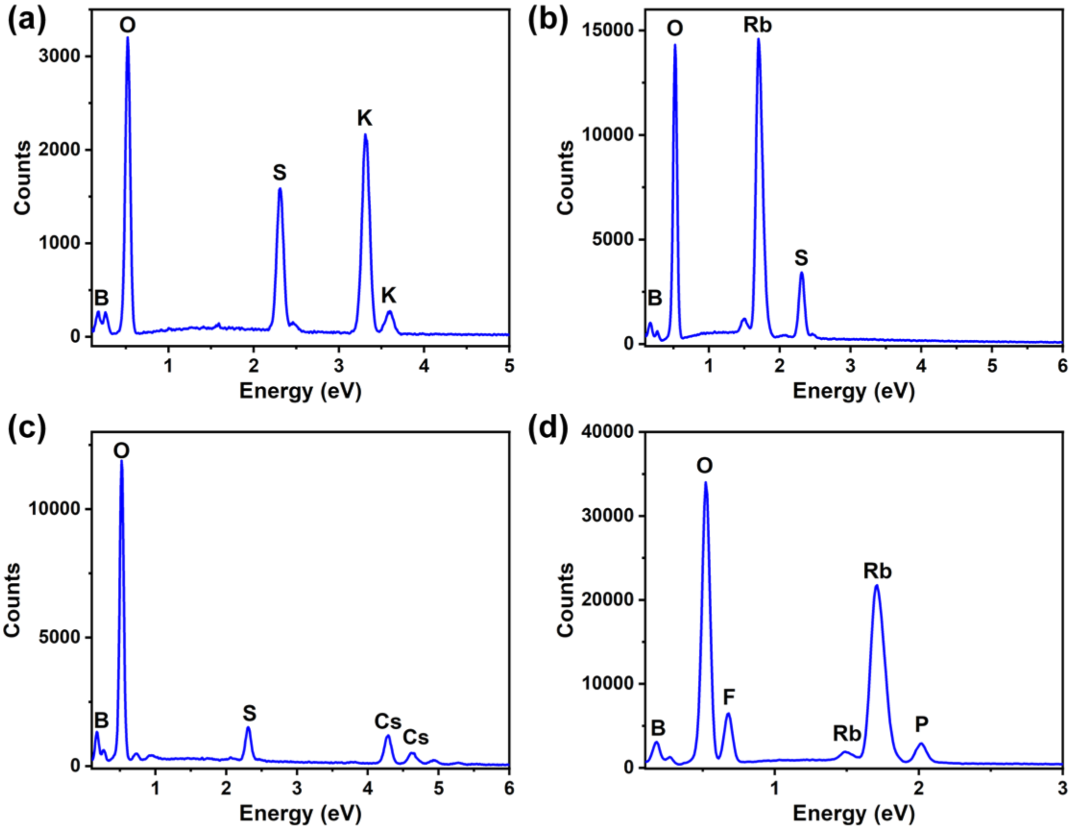


**Figure S5.** Energy dispersive X-ray spectroscope (EDS) analysis for $K_2B_4SO_{10}$ (a), $Rb_2B_4SO_{10}$ (b) $Cs_2B_4SO_{10}$ (c), and $Rb_3B_{11}PO_{19}F_3$ (d).

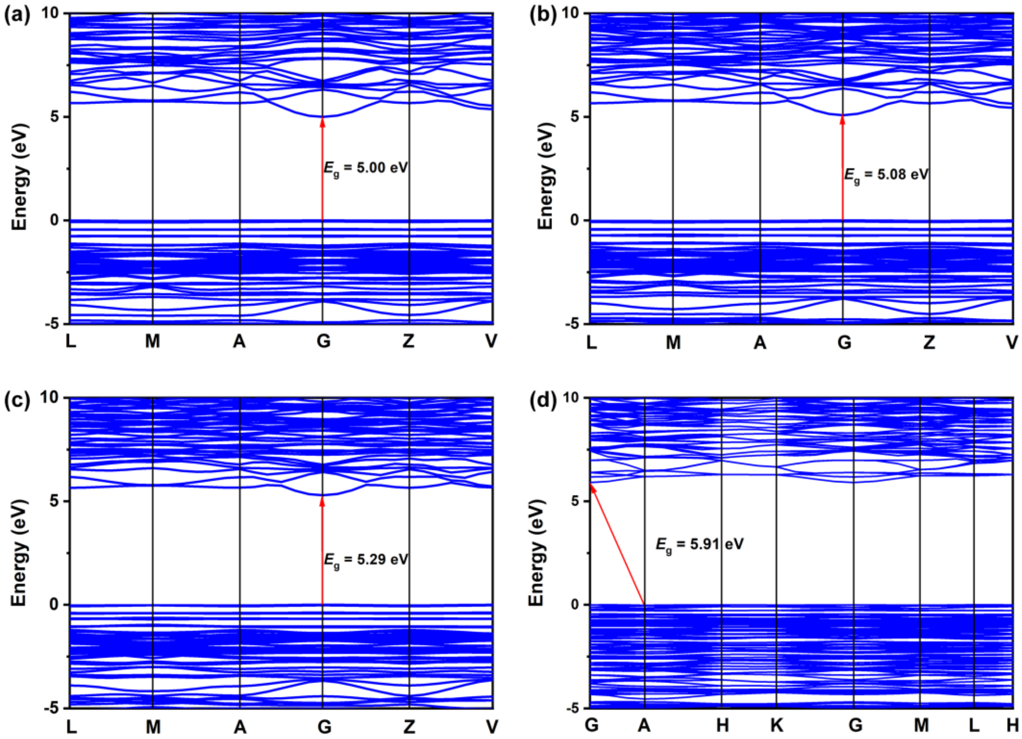


**Figure S6.** The calculated band structures of $M_2B_4SO_{10}$ (M = K, Rb, and Cs) (a-c) and $Rb_3B_{11}PO_{19}F_3$ (d) based on GGA.

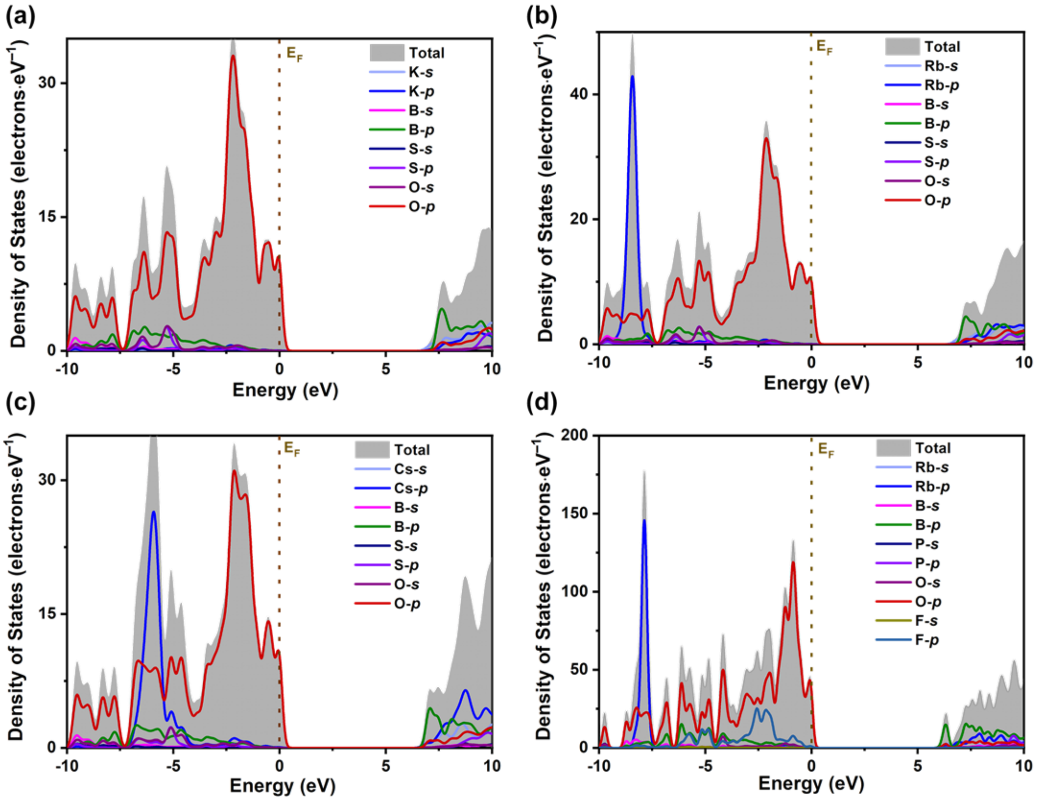


**Figure S7.** Total and partial density of states plots for $K_2B_4SO_{10}$ (a), $Rb_2B_4SO_{10}$ (b), $Cs_2B_4SO_{10}$ (c), and $Rb_3B_{11}PO_{19}F_3$ (d). The Fermi levels (EF) are marked.

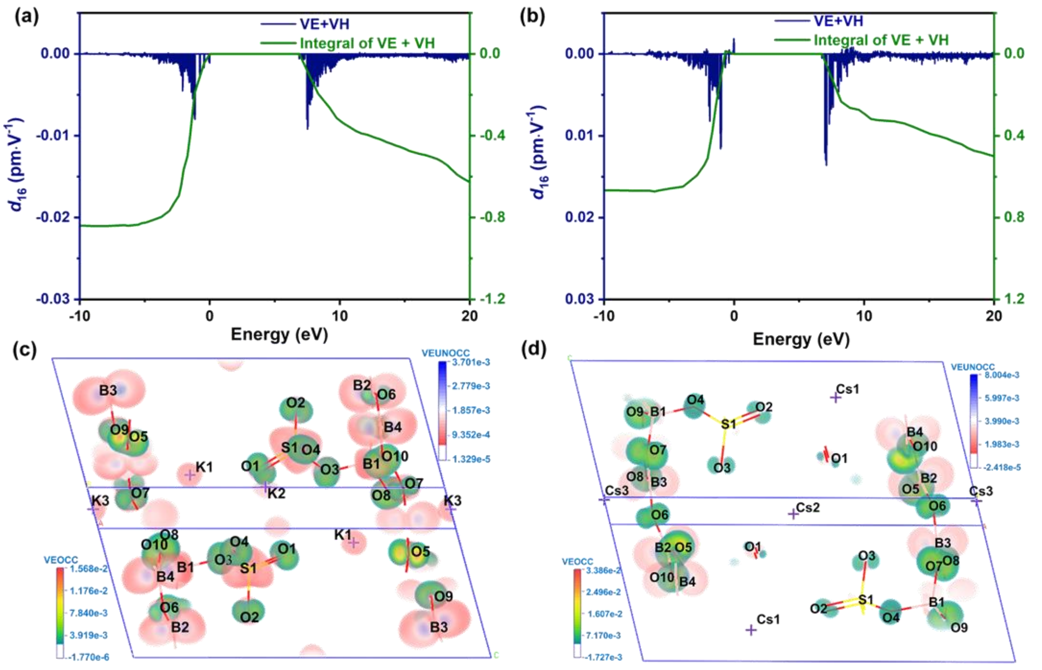


**Figure S8.** Band-resolved analysis of $K_2B_4SO_{10}$ (a) and $Cs_2B_4SO_{10}$ (b). SHG density maps of occupied and unoccupied orbitals in the virtual electron (VE) process of $d_{16}$ in $K_2B_4SO_{10}$ (c) and $Cs_2B_4SO_{10}$ (d).

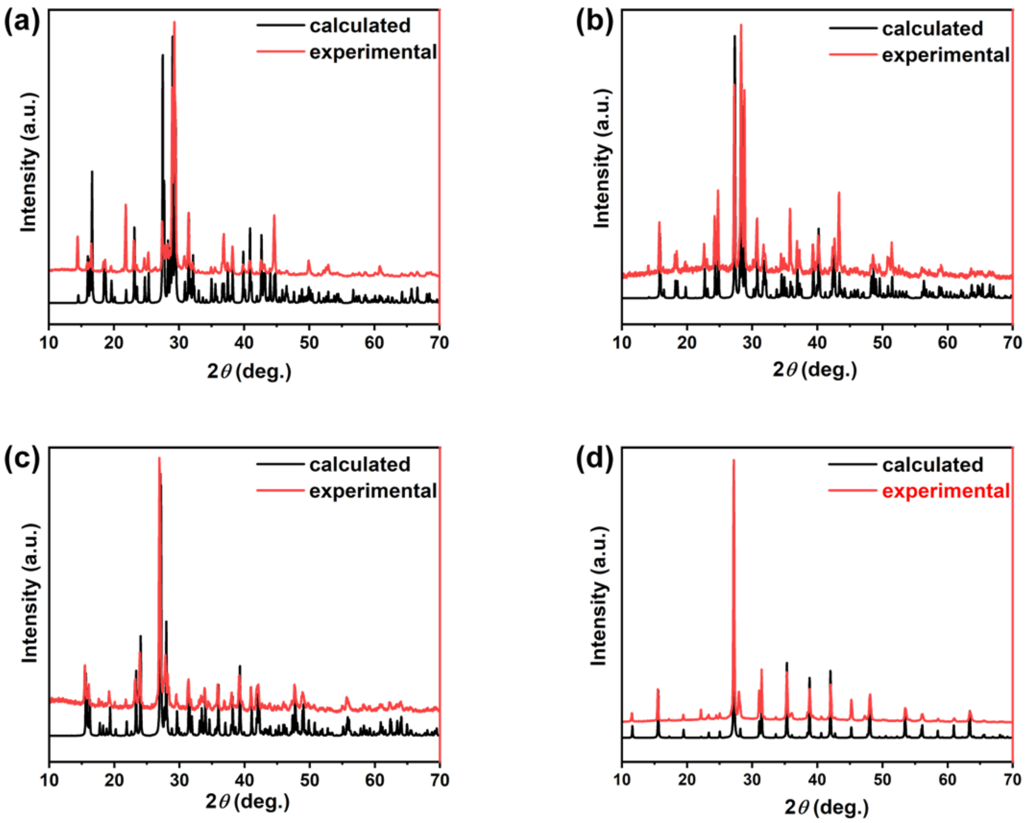


**Figure S9.** Experimental and calculated XRD patterns for $K_2B_4SO_{10}$ (a), $Rb_2B_4SO_{10}$ (b), $Cs_2B_4SO_{10}$ (c), and $Rb_3B_{11}PO_{19}F_3$ (d).

## REFERENCES


(1) SAINT Plus, Version 7.60A; Bruker Analytical X-ray Instruments, 2000.

(2) Sheldrick, G. M. Crystal Structure Refinement with SHELXL. *Acta Crystallogr. C* **2015**, *71*, 3−8.

(3) Lennartson, A., Optical Resolution and Racemisation of [Fe(acac)3]. Inorg. Chim. Acta 2011, 365, 451-453.

(4) Spek, A. L. Single-Crystal Structure Validation with the Program PLATON. *J. Appl. Crystallogr.* **2003**, *36*, 7−13.

(5) Kurtz, S. K.; Perry, T. T. A Powder Technique for the Evaluation of Nonlinear Optical Materials. *J. Appl. Phys.* **1968**, *39*, 3798−3813.

(6) Clark, S. J.; Segall, M. D.; Pickard, C. J.; Hasnip, P. J.; Probert, M. J.; K. Refson, M. C. First Principles Methods Using CASTEP. *Payne, Z. Kristallogr.* **2005**, *220*, 567.

(7) Perdew, J. P.; Burke, K.; Ernzerhof, M. Generalized Gradient Approximation Made Simple. *Phys. Rev. Lett.* **1996**, *77*, 3865.

(8) Filippi, C.; Singh, D. J.; Umrigar, C. J. All-Electron Local-Density and Generalized-Gradient Calculations of the Structural Properties of Semiconductors. *Phys. Rev. B*, **1994**, *50*, 14947−14951.

(9) Städele, M.; Moukara, M.; Majewski, J. A.; Vogl, P. Exact Exchange Kohn-Sham Formalism Applied to Semiconductors. *Phys. Rev. B*, **1999**, *59*, 10031−10043.

(10) Aversa, C.; Sipe, J. E. Nonlinear Optical Susceptibilities of Semiconductors: Results with a Length-Gauge Analysis. *Phys. Rev. B* **1995**, *52*, 14636.

(11) Rashkeev, S. N.; Lambrecht, W. R.; Segall, B. Efficient *ab initio* Method for the Calculation of Frequency-Dependent Second-Order Optical Response in Semiconductors. *Phys. Rev. B* **1998**, *57*, 3905.

(12) Lin, J.; Lee, M. H.; Liu, Z. P.; Chen, C. T.; Pickard, C. J. Mechanism for Linear and Nonlinear Optical Effects in $\beta$-$BaB_2O_4$ Crystals. *Phys. Rev. B* **1999**, *60*, 13380.

(13) Zhang, B. B.; Lee, M. H.; Yang, Z. H.; Jing, Q.; Pan, S. L.; Zhang, M.; Wu, H. P.; Su, X.; Li, C. S. Simulated Pressure-Induced Blue-Shift of Phase-Matching Region and Nonlinear Optical Mechanism for $K_3B_6O_{10}X$ (X = Cl, Br). *Appl. Phys. Lett.* **2015**, *106*, 031906.

(14) Lee, M. H.; Yang, C. H.; Jan, J. H. Band-Resolved Analysis of Nonlinear Optical Properties of Crystalline and Molecular Materials, *Phys. Rev. B* **2004**, *70*, 235110.

(15) Lo C. H. The Role of Electron Lone-Pair in the Optical Nonlinearity of Oxide, Nitride and Halide Crystals [D], Tamkang University, **2005**.

(16) Palik, E. D. Handbook of Optical Constants of Solids; Academic Press: New York, **1985**.

(17) Lei, B. H.; Yang, Z. H.; Pan, S. L. Enhancing Optical Anisotropy of Crystals by Optimizing Bonding Electron Distribution in Anionic Groups. *Chem. Commun.* **2017**, *53*, 2818−2821.
